# TRIP Steels: A Multiscale Computational Simulation and Experimental Study of Heat Treatment and Mechanical Behavior

**DOI:** 10.3390/ma13020458

**Published:** 2020-01-18

**Authors:** Ioanna Papadioti, Ilias Bellas, Maria-Ioanna T. Tzini, Peter I. Christodoulou, Nikolaos Aravas

**Affiliations:** 1 Department of Mechanical Engineering, University of Thessaly, 38334 Volos, Greece; papadioti@uth.gr (I.P.); iliasbellas@gmail.com (I.B.); margiannatz@gmail.com (M.-I.T.T.); pchristod@uth.gr (P.I.C.); 2 International Institute for Carbon Neutral Energy Research (WPI-I2CNER), Kyushu University, 744 Moto-oka, Nishi-ku, Fukuoka 819-0395, Japan

**Keywords:** TRIP steels, strain-induced transformation, retained austenite stability, phase-field modeling, finite element methods, homogenization, elasto-plasticity, composite materials, forming limit diagrams

## Abstract

A multiscale investigation of the microstructure and the mechanical behavior of TRIP steels is presented. A multi-phase field model is employed to predict the microstructure of a low-alloy TRIP700 steel during a two-stage heat treatment. The resulting stability of retained austenite is examined through the $M_{s}^{\sigma }$ temperature. The phase field results are experimentally validated and implemented into a model for the kinetics of retained austenite during strain-induced transformation. The kinetics model is calibrated by using experimental data for the evolution of the martensite volume fraction in uniaxial tension. The transformation kinetics model is used together with homogenization methods for non-linear composites to develop a constitutive model for the mechanical behavior of the TRIP steel. A methodology for the numerical integration of the constitutive equations is developed and the model is implemented in a general-purpose finite element program (ABAQUS). Necking of a bar in uniaxial tension is simulated and “forming limit diagrams” (FLDs) for sheets made of TRIP steels are calculated. The models developed provide an integrated simulation toolkit for the computer-assisted design of TRIP steels and can be used to translate mechanical property requirements into optimised microstructural characteristics and to identify the appropriate processing routes.

## 1. Introduction

**TR**ansformation **I**nduced **P**lasticity (TRIP) steels is a new generation of low alloy steels that exhibit an enhanced combination of strength and ductility. The microstructure of TRIP steels consists typically of ferrite, bainite, and retained austenite. Their remarkable mechanical properties are attributed to the TRIP phenomenon, i.e., the retained austenite transforms to martensite with plastic deformation. In the present work, a multiscale investigation of the microstructure and the mechanical properties of TRIP steels is presented. This investigation can be conducted for other steel grades applied in the automotive industry with the appropriate calibration.

The stabilization of retained austenite against martensitic transformation is a key criterion for the design of TRIP steels [1,2]. A common approach to stabilize the retained austenite, is to perform a two-stage heat treatment, consisting of intercritical annealing prior to isothermal bainitic transformation and quenching to room temperature [3,4]. During the isothermal bainitic transformation the remaining austenite is enriched with sufficient carbon and stabilized as retained austenite. Several studies were carried out in order to investigate and predict the microstructural evolution during intercritical annealing [5,6,7], bainitic isothermal transformation [8,9,10] and martensitic transformation [11].

The determination of retained austenite stability, expressed by the $M_{s}^{\sigma }$ temperature, and the description of retained austenite strain-induced transformation kinetics are essential in predicting the mechanical performance of TRIP steels. The chemical composition and grain size of retained austenite, as well as the stress triaxiality and the strength of the austenite matrix were considered to be the primary influencing factors on $M_{s}^{\sigma }$ [12,13,14,15]. Several models, which are reviewed by Samek et al. [16], were developed for the transformation kinetics of retained austenite. In addition, Haidemenopoulos et al. [17] took into account the effect of austenite particle size on transformation kinetics. Regarding the description of the mechanical behavior of TRIP steels, several constitutive models were developed [18,19,20]. However, these models disregard the effect of austenite particle size on transformation kinetics. Zhu et al. [21] and Shengyen [22] employed computational-based approaches for the design of TRIP steels, yet a multiscale integrated model is required for the investigation of the microstructure and the mechanical properties of the investigated steels. A brief description of each section of the present work is given below.

In Section 2, an integrated simulation model is employed to predict the microstructural characteristics of a cold rolled low-alloy TRIP700 steel subjected to a two-stage heat treatment. The heat treatment consists of intercritical annealing and an isothermal bainitic transformation at a lower temperature. A two-dimensional (2D) multi-phase field model (MPF) is developed for the simulation of the intercritical annealing process after deformation in a ferrite-pearlite initial structure. The concentration profiles, volume fractions of the phases, and microstructure obtained at the end of the intercritical annealing are implemented into the MPF model for the simulation of the isothermal bainitic treatment. The MPF model is used to determine the volume fractions and the average grain size of the phases as well as their chemical content in the resulting microstructure at the end of the heat treatment.

The experimental validation of the predictions for the heat treatment process is discussed in Section 3. The average grain size diameter of ferrite and retained austenite are determined using optical microscopy. The Saturation Magnetization (SM) technique is employed to evaluate the volume fraction of retained austenite. By applying the SS-TV-TT technique to determine the $M_{s}^{\sigma }$ temperature, the austenite stability is measured.

The austenite stability, which influences the kinetics of transformation plasticity, is studied in Section 4 by using the model of Haidemenopoulos and Vasilakos [15] for the stability of dispersed austenite in low alloy steels. This model predicts the effects of several parameters on austenite stability. An attempt is made to distinguish the effects of individual parameters on austenite stability. The predictions of the model are consistent with the experimental results for the $M_{s}^{\sigma }$ temperature. Also, in Section 4, the model of Haidemenopoulos et al. [17] is employed to predict the evolution of the volume fraction of martensite during the strain-induced transformation of retained austenite. The microstructural characteristics calculated from the simulation of the heat treatment are used as input to this model, which takes into consideration the chemical composition, the austenite particle size, temperature, and stress state. The calibration of the model is based on experimental data for the evolution of the martensite volume fraction in uniaxial tension tests.

In Section 5, the aforementioned transformation kinetics model is used together with non-linear homogenization methods of Ponte Castañeda and co-workers [23,24,25] to develop constitutive equations for the mechanical behavior of TRIP steels. The methodology of Papadioti et al. [26], in which TRIP steels are treated as multi-phase (composite) materials, is used to estimate the effective yield function and the average stress and strain fields in the constituent phases.

A methodology for the numerical integration of the resulting constitutive equations in the context of a displacement driven finite element formulation is presented in Appendix A and Appendix B. The model is implemented in the ABAQUS general-purpose finite element code, one-element finite element calculations for the problem of uniaxial tension are performed, and the simulation results are compared with experimental data in Section 6. In Section 7, the constitutive model is employed for the study of necking under uniaxial tension and forming limit diagrams (FLDs) are also calculated.

The scope of the present work, is to provide an integrated simulation toolkit, which will be used for the design of TRIP steels. Starting with the simulation of the microstructure evolution with respect to the heat treatment parameters, the chemical composition and the grain size of retained austenite are calculated. These results are used as an input for the description of the stability of retained austenite and the martensite volume fraction formed as a function of plastic strain in TRIP steels. After the experimental validation of the heat treatment predictions and the $M_{s}^{\sigma }$ temperature, a constitutive model is developed for the mechanical behavior of TRIP steels with respect to the processing parameters and microstructural features. The integrated model presented in this work links composition, processing, microstructure and mechanical behavior and makes it a potential design tool for the development of optimized TRIP steels.

Throughout the text, standard notation is used, while boldface symbols correspond to tensors, the orders of which are indicated by the context. A fixed Cartesian coordinate system with base vectors $e_{i}\;\;\left(i=1,2,3\right)$ is used for the expression of all tensor components, and Greek indices take the values 1 and 2. The summation convention is applied for repeated latin and Greek indices $\left(c_{\alpha }\;d_{\alpha }\equiv \sum_{i=1}^{2}c_{\alpha }\;d_{\alpha }\right)$. A superscript *T* denotes the transpose, and a superposed dot the material time derivative. Let (A, B) be second-order tensors and (C,D) fourth-order tensors; the following products are used in the text ${\left(A\;\cdot \;B\right)}_{ij}=A_{ik}\;B_{kj}$, $A:B=A_{ij}\;B_{ij}$, ${\left(A\;B\right)}_{ijkl}=A_{ij}\;B_{kl}$, ${\left(C:A\right)}_{ij}=C_{ijkl}\;A_{kl}$, ${\left(A:C\right)}_{ij}=A_{kl}\;C_{klij}$. $A:C:B=A_{ij}\;C_{ijkl}\;B_{kl}$, and ${\left(C:D\right)}_{ijkl}=C_{ijpq}\;D_{pqkl}$. The inverse $C^{-1}$ of a fourth-order tensor C that has the “minor” symmetries $C_{ijkl}=C_{jikl}=C_{ijlk}$ is defined so that $C:C^{-1}=C^{-1}:C=I$, where I is the symmetric fourth-order identity tensor with Cartesian components $I_{ijkl}=\frac{1}{2}\left(\delta_{ik}\;\delta_{jl}+\delta_{il}\;\delta_{jk}\right)$, $\delta_{ij}$ being the Kronecker delta.

## 2. Materials and Modeling of the Heat Treatment

An integrated process chain model is employed for the prediction of the microstructural features of a cold rolled (CR) TRIP700 steel during a two-stage heat treatment, which consists of intercritical annealing prior to isothermal bainitic transformation at a lower temperature. TRIP 700 is a well-established TRIP steel, this is why it was used as a model steel in the investigation. This investigation can be carried out for other steel grades used in the automotive industry with the appropriate calibration. The chemical composition of the CR-TRIP700, provided by voestalpine Stahl GmbH Linz, is described in Table 1. After cold-rolling, the aim is to form a 50%-50% ferrite-austenite microstructure during intercritical annealing in the first stage and to stabilize the retained austenite through an isothermal bainitic transformation in the second stage.

### 2.1. Selection of Heat Treatment Parameters

An integrated process chain model is developed using multi-phase field modeling. The thermodynamic calculations, required to set the bounds of the process, are performed using the Thermo-Calc software [27] and the TCEF6 database for ferrous alloys. The limits for the intercritical annealing are $A_{1}=693.4{}^{\circ}C$ and $A_{3}=917.8{}^{\circ}C$, and the cementite solvus temperature is $A_{cem}=716.8{}^{\circ}C$. During intercritical annealing, the target volume fraction of austenite is 50%. Thermodynamic calculations show that the intercritical annealing should take place above 808.3°C. An intercritical annealing temperature at 890°C is selected with a heating rate of 15°C/s and an intercritical annealing time of 60 s, followed by an isothermal bainitic treatment consisting of cooling with a rate of 60°C/s and an isothermal holding at 400°C for 120 s.

### 2.2. Methodology

The multi-phase field model (MPF), developed by Steinbach et al. [28,29,30,31] and implemented in MICRESS [32], is used for the description of microstructure evolution during intercritical annealing and isothermal bainitic treatment. The adopted approach takes into account the effect of grain boundary curvature and allows the determination of the spatial distribution of phases and their grain size [33].

#### 2.2.1. Stage I—Intercritical Annealing

For the simulation of the intercritical annealing process after deformation of a ferrite-pearlite initial structure, a 2D MPF model is employed. Since the heating rate is 15°C/s, it is considered that ferrite recrystallization is completed and austenite nucleates mainly in pearlite [5,7,34,35,36]. A very small amount of austenite nuclei is allowed to form at ferrite grain boundaries, when it is thermodynamically possible. Spheroidization of pearlite is neglected. A sufficiently large domain size is used in the calculations, so that there are no significant statistical differences in the results [37]. However the description of a fine lamellar pearlite structure is not possible in this study, since a smaller domain size is required [38,39]. For this reason, pearlite is modelled as an effective phase with uniform eutectoid composition of 0.86% wt. carbon and an equilibrium phase fraction of 22.9%. The simulations are carried out in two steps. In the *first step*, reheating from the $A_{1}$ temperature to the selected intercritical annealing temperature takes place for a time period sufficient for complete dissolution of pearlite. A rapid pearlite to austenite transformation occurs and austenite is assumed to inherit the carbon content of pearlite; the substitutional elements are assumed to be immobile. The growth kinetics of the phases and the redistribution of the alloying elements are described by non-partitioning local equilibrium conditions (NPLE). Austenite is allowed to nucleate at ferrite grain boundaries. The *second step* starts after the complete dissolution of pearlite. During the second step, ferrite transforms to austenite and the austenite formed in the first step continues to grow. The ferrite to austenite phase transformation takes place under ortho-equilibrium conditions.

The domain size is 85.5μm×40.5μm. A grid of square cells with sides δx=0.15μm is used in the calculations. The interface thickness is set to 0.6μm. The initial structure is constructed randomly and periodic boundary conditions are applied. The Thermo-Calc TCEF6 and MOB2 databases are used to derive diffusion data. The same diffusion coefficients are used for pearlite and ferrite. Thermodynamic data for the ferrite-pearlite and pearlite-austenite interactions are obtained using a linearized phase diagram; the TCEF6 database is used to get the corresponding data for the ferrite-austenite interaction. The local equilibrium temperature is set according to the linearized phase diagram at 744°C, where the equilibrium carbon content is 0.86% wt. and 0.0063% wt. in pearlite and ferrite respectively. Manganese and aluminum are considered immobile during the first step of the simulations; their nominal compositions are selected as the equilibrium concentrations in the phases at 744°C.

The interfacial energies $\sigma_{ij}$ for the phase interactions take the constant values listed in Table 2 [35,37]. The interface mobility $\mu_{ij}$ between phases *i* and *j* is considered to be temperature-dependent:

(1) $$\mu_{ij}=\mu_{ij}^{o}\;\exp\left(-\frac{Q_{ij}}{R\;T}\right),$$

where $\mu_{ij}^{o}$ is the pre-exponential mobility factor, *R* the gas constant, and $Q_{ij}$ the activation energy for grain boundary migration. The pre-exponential factor is written in the form

(2) $$\mu_{ij}^{o}=\frac{v_{D}}{k\;T}\;d_{ij}^{4},$$

where $d_{ij}$ is the interatomic distance between phases *i* and *j*, $v_{D}$ the Debye frequency of the parent phase, and *k* the Boltzmann constant. The mobility parameters for the phase interactions $Q_{ij}$ and $d_{ij}$ are taken from the literature [40,41] and are listed in Table 2.

In the first step of simulations, austenite nucleates within pearlite and grows until complete dissolution of pearlite. Nucleation of austenite within pearlite is assumed to be instantaneous (site-saturated). The growth of austenite is controlled by carbon diffusion in austenite and the partitioning of substitutional atoms in ferrite [42]. It is assumed that the critical surface area of a nucleus is 1 $\mu m^{2}$. The number of austenite potential nucleation sites in pearlite is $N_{n}=793$ and equals the product of the pearlite phase fraction and the domain size divided by the nucleus surface area. Austenite nucleation is allowed to occur at ferrite grain boundaries both in the first and the second step, if it is thermodynamically possible. The expression that defines the heterogeneous nucleation rate of austenite in ferrite ${\dot{N}}_{S}$ is based on classical nucleation theory under steady state conditions [35] and is of the form

(3) $${\dot{N}}_{S}\left(t\right)=N_{n}\left(t\right)\frac{k\;T}{h}\exp\left[-\frac{1}{k\;T}\left(\frac{\psi }{\Delta g_{v}^{2}}+Q_{D}\right)\right],$$

where $N_{n}\left(t\right)$ is the number of potential nucleation sites as a function of time, a superposed dot denotes derivative with respect to time, ψ is a constant related to the geometry of the nucleus (shape) and the interfacial properties for the nucleus and the matrix, $\Delta g_{v}$ is the driving force for nucleation of austenite in ferrite per unit volume and was calculated using Thermo-Calc, *h* and *k* are the Planck and Boltzmann constants accordingly, and $Q_{D}$ is the activation energy for nucleation, which is approximately equal to the activation energy of iron self-diffusion in ferrite and takes the value of $3.9\times 10^{-19}\;J$. The value of $\psi_{a}=4.76\times 10^{-10}\;J^{3}/m^{6}$ is used for nucleation on ferrite grain boundaries (Savran [40]). The value of $N_{n}$ is kept constant in the calculations and its value equals the product of ferrite grain boundary perimeter times the interface thickness divided by the nucleus surface area. In the 2D simulation domain, the number of austenite potential nucleation sites is $N_{n}=2436.6\;\mu m\times 0.6\;\mu m/1\;\mu m^{2}=1462$. A random orientation of austenite nuclei is considered with isotropic austenite and ferrite grain growth.

#### 2.2.2. Stage II—Isothermal Bainitic Treatment

Bainitic ferrite is assumed to form by diffusional nucleation and displacive transformation [43]. The nucleation and growth of the entire sheaf is considered instead of the formation of sub-units. Diffusion of the alloying elements is allowed. Since excess Si and Al content in steels can suppress carbide precipitation, cementite precipitation is not considered neither in bainite nor in austenite. Plastic strains developed during the sub-unit growth are not accounted for and the plate shape morphology of bainitic ferrite is acquired artificially by using the anisotropic interfacial properties. Accordingly, the model cannot account the incomplete reaction phenomenon of bainite due to dislocation debris. Solute drag effect of substitutional atoms are ignored in the interface kinetics and Zener pinning forces due to the precipitation of carbonitrides are not included in this study.

The concentration profiles, volume fractions of the phases, and microstructure obtained at the end of the intercritical annealing are implemented into the MPF model for the simulation of the isothermal bainitic treatment. The same domain size and the interface thickness are used in the calculations. The thermodynamic data for the interaction of ferrite and austenite are derived by coupling the multi-phase field calculations with the Thermo-Calc TCEF6 and MOB2 databases. To simplify the simulations, no phase interaction between ferrite and bainite is considered. For the description of austenite-bainite phase interaction, a linearized phase diagram is calculated at the local equilibrium temperature of 744°C. The same diffusion coefficients, interfacial energies, and interface mobilities are used both for ferrite and bainitic ferrite phases. A value of $\sigma^{\gamma -a^{\prime }}=0.2\;J/m^{2}$ [44] is used for the interfacial energy between austenite and bainite; the interface mobility at 400°C is set to $\mu^{\gamma -a^{\prime }}=9\times 10^{-9}\;{\mathrm{cm}}^{4}/\left(J\;s\right)$.

Bainitic ferrite is modelled as an anisotropic phase with tetragonal symmetry. In TRIP steels there is a Kurdjumov–Sachs (KS) orientation relationship that describes the relative orientation of retained austenite and bainitic ferrite [45]. It is assumed that the misorientation angle between bainite grains and parent austenite grains follows a KS relationship with a tolerance angle of 10°. The transformation model of Bhadeshia [46] is used for the calculation of the bainite start temperature. The required driving force per mole for nucleation in austenite $\Delta g_{v}$ and the diffusionless driving force for bainite formation $\Delta G^{\gamma \to a^{\prime }}$, which is regarded as the Gibbs free energy difference of ferrite and austenite, are calculated using Thermo-Calc. Bainite start temperature is calculated as $B_{S}=616.8{}^{\circ}C$. The nucleation rate of bainitic ferrite is calculated according to equation (3), where the activation energy for self-diffusion of carbon in austenite $Q_{D}=142.1\;\mathrm{kJ}/\mathrm{mol}$ is used [7]. For bainite nucleation at austenite grain boundaries the value $\psi_{a^{\prime }}=6.33\times 10^{-15}\;J^{3}/{\mathrm{mol}}^{2}$ is used [47]. The number of bainite potential nucleation sites is represented by the total surface area of austenite grain boundaries, which is the product of the austenite grain boundary perimeter times the interface thickness divided by the nucleus surface area, and takes the value $N_{n}=2573.3\;\mu m\times 0.6\;\mu m/1\;\mu m^{2}=1544$.

### 2.3. Simulation Results of the Heat Treatment

**Figure 1:**
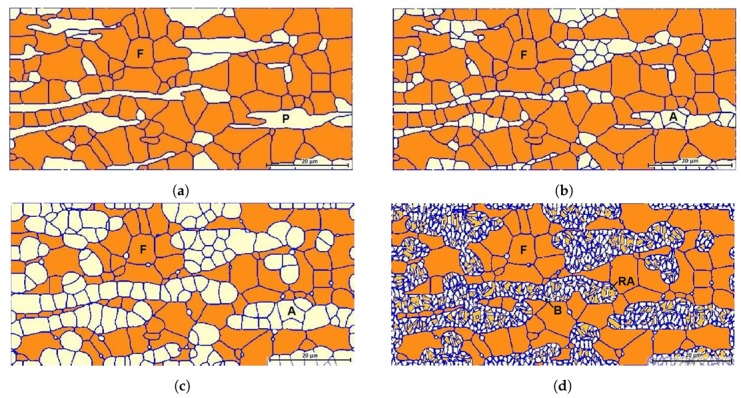
Microstructures (**a**) at the beginning of the intercritical annealing, (**b**) after the complete dissolution of pearlite, (**c**) at the end of the intercritical annealing, and (**d**) at the end of the isothermal bainitic treatment. Symbols *P*, *F*, and *A* denote pearlite, ferrite, and austenite grains, whereas *RA* and *B* correspond to retained austenite and bainitic ferrite.

Figure 1a shows the initial structure, which consists of 99 recrystallized ferrite grains and 36 elongated pearlite grains. The initial average grain size is 5.65 μm for pearlite and 6.24 μm for ferrite, where equivalent circular mean diameters are considered. The microstructure after the complete dissolution of pearlite is depicted in Figure 1b. Symbols *P*, *F*, and *A* denote pearlite, ferrite and austenite grains respectively. The simulation time required for the complete dissolution of pearlite was 9 s at 828 °C. The formed austenite nuclei in pearlite are 164, whereas no austenite nuclei were observed at ferrite grain boundaries during the first step of simulations.

In the second step, the formed austenite grows at the expense of ferrite, while some austenite nuclei grow at ferrite grain boundaries until the end of intercritical annealing at 890 °C. After 60 s of intercritical annealing, a 51% volume fraction of austenite was achieved; the corresponding microstructure is shown in Figure 1c. The microstructure at the end of the isothermal bainitic treatment at 400 °C for 120 s is presented in Figure 1d. Symbols RA and *B* correspond to the retained austenite and the bainitic ferrite.

**Figure 2:**
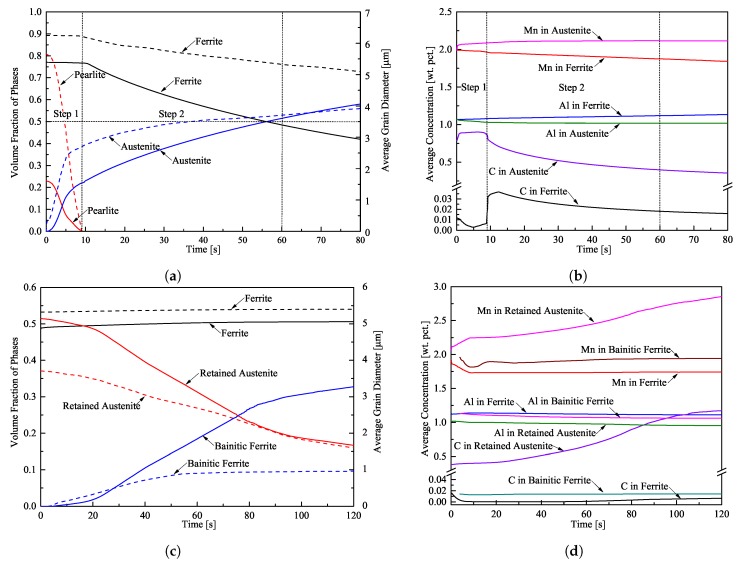
Temporal variation of (**a**) the volume fraction (solid lines) and average grain diameter (dashed lines) of ferrite, pearlite, and austenite, (**b**) average concentration of C, Al, and Mn in ferrite and austenite during intercritical annealing at 890 °C, (**c**) the volume fraction (solid lines) and average grain diameter (dashed lines) of ferrite, retained austenite, and bainitic ferrite, and (**d**) average concentration of C, Al, and Mn in ferrite, retained austenite, and bainitic ferrite during isothermal bainitic treatment at 400 °C.

The evolution of the volume fraction of the phases and the average grain diameter of pearlite, ferrite, and austenite during intercritical annealing are depicted in Figure 2a. Continuous lines correspond to volume fractions and dashed lines indicate the average grain size. The vertical dotted lines indicate the end of the first step and the time (∼60 s) at which 51% volume fraction of austenite was achieved; the horizontal dotted line marks the targeted 50%-50% ferrite-austenite microstructure. It should be noted that austenite grows faster in pearlite during the first step; the rate of austenite growth in ferrite is slower in the second step. The average grain diameters of ferrite and austenite at the intercritical annealing time of t=60 s are 5.31 μm and 3.72 μm respectively.

The average concentration of carbon, aluminum, and manganese in ferrite and austenite are plotted versus time in Figure 2b. At the beginning of the second step, there is a carbon enrichment in ferrite. However, after 13 s, when the intercritical temperature of 890 °C has been reached, the average carbon content in ferrite starts decreasing due to the decrease of the maximum soluble amount of carbon in ferrite at this temperature. This temporal carbon enrichment in ferrite is favourable for the austenite formation at the ferrite grain boundaries. Carbon depletion in austenite is still observed due to the increase of austenite phase fraction. Regarding the substitutional atoms, there is manganese depletion and aluminum enrichment in ferrite.

The evolution of the volume fractions and the average grain diameter of ferrite, retained austenite, and bainitic ferrite during the isothermal bainitic treatment are shown in Figure 2c. Continuous lines correspond to volume fractions and dashed lines indicate the average grain diameter of the phases. Bainitic ferrite starts to form after 6 s of cooling and isothermal holding. When bainite is formed, the austenite shrinks and ferrite grains grow slowly. After 120 s, the resulting microstructure in the new TRIP steel consists of 50.7% ferrite, 16.6% retained austenite, and 32.7% bainitic ferrite, and their average grain diameters are 5.39 μm, 1.62 μm, and 0.96 μm accordingly. For the rest of the paper, the term “***TRIP steel A***” is used to refer to this microstructure.

The average concentration of carbon, aluminum, and manganese in ferrite, retained austenite, and bainitic ferrite are depicted in Figure 2d. During bainite transformation, carbon and manganese are ejected from bainite in austenite, while aluminum diffuses into bainite and ferrite until equilibrium is reached. The carbon content in retained austenite after 120 s is 1.17% wt.

## 3. Experimental Study

### 3.1. Heat Treatment

A TRIP700 steel which consists of ferrite, retained austenite, bainitic ferrite with traces of martensite, was employed for the experimental validation of the heat treatment process presented in Section 2. An austenitization process was performed above the $A_{3}$ temperature, at 950 °C, to erase its current microstructure. After the austenitization, the material is air cooled to room temperature, and a mixed microstructure consisting of ferrite, pearlite, and a small amount of bainitic ferrite was formed. It should be noted that for the simulation of the heat treatment process, an initial deformed ferrite-pearlite structure was assumed prior to the intercritical annealing process. This assumption leads to different initial microstructures for the intercritical annealing between the experimental study and the phase-field simulations. As a result, minor deviations between experimental data and simulation results, regarding the morphology and the phase volume fractions, are observed.

**Figure 3:**
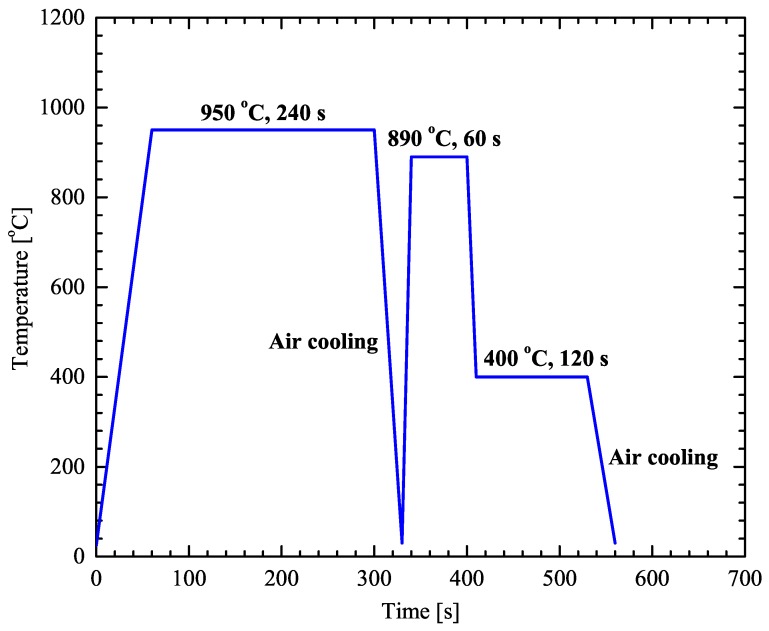
Heat treatment process of studied material.

The heat treatment process is presented schematically in Figure 3.

The heating process consists of three steps that include austenitization, intercritical annealing, and austempering for bainitic transformation. Six specimens with dimensions 120 mm ×100 mm ×1.5 mm were preheated in a front-load furnace, at 400 °C for 60 s and then directly inserted in a second furnace for the austenitization process at 950 °C for 240 s and air cooled to room temperature. Oxidation products from the surface of the samples were carefully removed by grinding with emery paper.

Intercritical annealing and austempering treatments were performed in furnaces with molten salt baths using GS750 and AS140 salts respectively. Following the austenitization process the samples were firstly preheated for 60 s at 400 °C and annealed at 890 °C for 60 s. Subsequently the samples cooled down to 400 °C for 120 s and air cooled to room temperature.

### 3.2. Evaluation of the $M_{s}^{\sigma }$ Temperature

**Figure 4:**
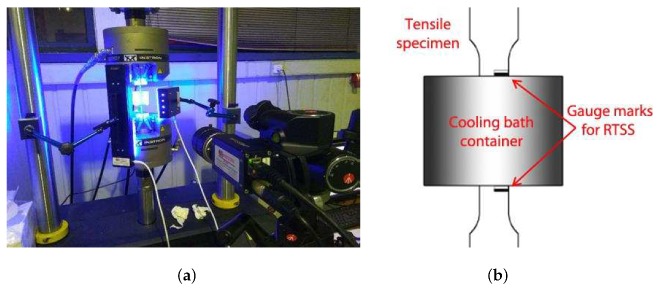
Experimental setup for the evaluation of $M_{s}^{\sigma }$ temperature. (**a**) RTSS Video-extensometersetup, (**b**) Graphical representation of specimen setup.

The stability of retained austenite (RA) was evaluated by measurements of the $M_{s}^{\sigma }$ temperature, which defines the critical boundary between the stress-assisted and strain-induced martensitic transformation in TRIP steels. $M_{s}^{\sigma }$ was determined experimentally with the SS-TV-TT technique [48,49] using the experimental setup shown in Figure 4. Loading-unloading was carried out in an INSTRON 8801 servo-hydraulic machine at a constant crosshead velocity of 0.5 mm/min, while a Real Time Strain Sensor (RTSS) video-extensometer was used to measure the longitudinal tensile strains during testing (Figure 4). Testing at low temperatures was performed using a plastic container with cooling bath attached at the gauge length area of the specimen. The desired temperatures, ranging from 20 °C to −30 °C, were achieved using appropriate concentrations of water, salt, ice, acetone, dry ice, and ethylene-glycol in the cooling bath.

**Figure 5:**
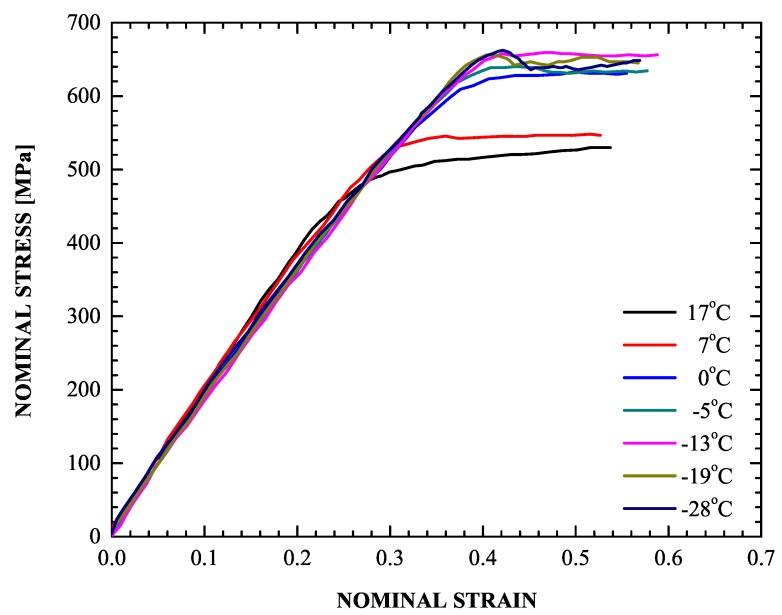
Stress – strain response under uniaxial tensile testing with decreasing temperature.

The $M_{s}^{\sigma }$ temperature was estimated from the stress-strain behavior during successive loading of the material up to yielding with decreasing temperature. The stress-strain curves obtained are depicted in Figure 5. They reveal an onset of stress relaxation at −19 °C, a result of the volume expansion accompanying the martensitic transformation phenomenon, i.e., the $M_{s}^{\sigma }$ temperature is found to be −19 °C, meaning that below that temperature martensitic transformation can only be stress-assisted; above −19 °C, strain-induced martensitic transformation takes place when austenite deforms plastically at temperatures below an upper limit $\left(M_{D}\right)$.

### 3.3. Microstructural Characterization

The microstructure after heat treatment was assessed using optical microscopy. Standard metallographic techniques of grinding and polishing were used in mounted sample. Consecutively, a stepped color tint-etching procedure was applied to reveal the microstructure. The sample was first etched with a 3% Nital solution for 3–4 s, and then with a 10% ${\mathrm{Na}}_{2}S_{2}O_{5}$ solution for 60 s. After etching in both solutions, the specimen was thoroughly washed with water, immersed in ethanol, and dried in hot air.

The average grain diameter of retained austenite (RA) and ferrite were estimated using an image analysis software [50]. Since there is a large dispersion of phases, several micrographs were taken at different depths. This method is suitable for structures with high grain shape irregularity [50]. Approximately 1000 retained austenite and 100 ferrite grains were used for the quantification of the above microstructural characteristics.

**Figure 6:**
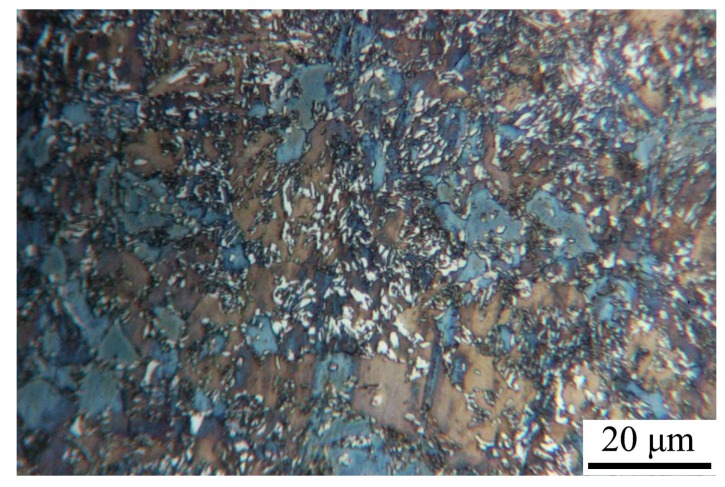
Micrograph of the TRIP material.

The RA-white colour, ferrite-blue/straw brown, and bainite-dark, microstructural characteristics are displayed in Figure 6, revealing a ferritic-bainitic matrix with a fine dispersion of RA. The average size of RA particle was 0.59 μm, while the average grain size of ferrite was 6.19 μm.

### 3.4. Retained Austenite Measurements

The %RA volume fraction in bulk material was measured with the Saturation Magnetization (SM) technique from small extracted samples of dimensions 14 mm ×3.5 mm. The SM measurements were performed in the Steel Division Department of voestalpine in Austria. Detailed description of the performed technique is presented in [51,52].

The advantages of this method are the ability to measure the entire volume of the specimen, the good repeatability [52], and the high precision compared to other methods [53] employed for the determination of the RA volume fraction. However, small uncertainties in the measurements may occur due to the existence of alloying elements, which can affect the saturation magnetization. For this reason, certain scatter in RA volume fraction measurements should always be taken into consideration.

An initial %RA volume fraction of 15.9% was measured, which is in a good agreement with the value of 16.6% calculated from the simulation of the heat treatment described in Section 2.3.

## 4. Stability and Transformation Kinetics of Retained Austenite

A thorough investigation regarding the stability of dispersed austenite in TRIP steel A is conducted based on the work of Haidemenopoulos and Vasilakos [15]. The $M_{s}$ temperature is used for the characterization of austenite stability against transformation on cooling, whereas the $M_{s}^{\sigma }$ temperature for the stability of dispersed austenite against mechanically induced transformation. During the isothermal bainitic transformation, the retained austenite is chemically enriched in carbon and manganese [54]. Primarily, the carbon content is a strong stabilizer of the retained austenite, leading to suppression of the $M_{s}$ temperature. However, a mechanically induced martensitic transformation can take place in dispersed austenite by strain-induced or stress-assisted nucleation [55,56]. A good combination of all the interrelated factors, which have a strong stabilizing influence on the kinetics of the mechanically induced transformation, is required so as to achieve the maximum TRIP effect. The aforementioned interrelation makes it difficult to separate the individual effect of each factor on austenite stability. This model is used to predict the $M_{s}^{\sigma }$ temperature and to identify and quantify the primary stabilizing factors in the TRIP steel. The model predictions ($M_{s}^{\sigma }$ temperature) are also compared with the experimental data obtained with the SS-TV-TT technique as described in Section 3.2. Furthermore, the model developed by Haidemenopoulos et al. [17] is employed to predict the evolution of the strain-induced martensitic transformation kinetics in the TRIP steel.

### 4.1. Methodology for the Calculation of $M_{s}^{\sigma }$

In the present work, the $M_{s}^{\sigma }$ temperature is chosen as the single parameter characterizing the stability of dispersed austenite particles in the TRIP microstructure. A variation of the methodology developed by Haidemenopoulos and Vasilakos [15] is used for the calculation of $M_{s}^{\sigma }$. According to the Olson-Cohen theory of heterogeneous martensitic nucleation [57,58,59], the dissociation of an existing defect serves as a potential nucleation site for the formation of a martensitic embryo. The energy per unit area $\gamma_{f}\left(n\right)$$\left(J/m^{2}\right)$ of an embryo with a thickness of *n* crystal planes is

(4) $$\gamma_{f}\left(n\right)=n\;\rho \;\left(\Delta G_{ch}+E_{str}+W_{f}\right)+2\;\gamma_{s},$$

where ρ is the atomic density in the close-packed fault plane $\left(\mathrm{mol}/m^{2}\right)$, $\Delta G_{ch}$(J/mol) the thermodynamic driving force for martensitic transformation, $E_{str}$(J/mol) the elastic strain energy related to the distortions in the interface plane of the fault structure, $W_{f}$(J/mol) the frictional work of interfacial motion accompanying the dissociation process, and $\gamma_{s}$$\left(J/m^{2}\right)$ the fault/matrix interfacial energy. The thickness *n* refers to the number of crystal planes forming the defect and its value can be interpreted as a potency measure of the nucleation site. Barrierless dissociation of an existing defect occurs when its thickness *n* is such that $\gamma_{f}\leq 0$. The condition $\gamma_{f}\left(n^{*}\right)=0$ defines the critical thickness $n^{*}$ of a defect:

(5) $$n^{*}=-\frac{2\;\gamma_{s}}{\rho \;\left(\Delta G_{ch}+E_{str}+W_{f}\right)}.$$

If the thickness *n* of the defect is greater than or equal to $n^{*}$, martensitic transformation takes place at that defect. The critical nucleation thickness of a defect $n^{*}$ depends on temperature through $\Delta G_{ch}$ and on the chemical enrichment of the austenite, which affects $\Delta G_{ch}$ and $W_{f}$.

To take into account the stress-dependence of $n^{*}$, the mechanical driving force $\Delta G_{\sigma }$(J/mol) is introduced in the denominator of Equation (5). Then, the total driving force is $\Delta G=\Delta G_{ch}+\Delta G_{\sigma }$, so that Equation (5) yields

(6) $$n^{*}\left(T,X,\sigma \right)=-\frac{2\;\gamma_{s}}{\rho \;\left[\Delta G_{ch}\left(T,X\right)+\Delta G_{\sigma }\left(\sigma \right)+E_{str}+W_{f}\left(X\right)\right]},$$

where $\Delta G_{\sigma }\left(\sigma \right)$ is a function of the stress tensor σ, *T* is temperature, and *X* denotes dependence on chemical composition. The last equation is based on the assumption that the orientation of the operative nucleation sites is optimum for maximum interaction with the applied stress (Patel and Cohen [60]). At the other extreme of a random distribution of nucleation sites, the term $\Delta G_{\sigma }$ should be replaced by $\Delta G_{\sigma }/3$ in (6) (Olson et al. [61]).

In isotropic materials, $\Delta G_{\sigma }\left(\sigma \right)$ is in general a function of the von Mises equivalent stress $\sigma_{e}$, the “stress triaxiality” ∑, and the “Lode angle” θ:

(7) $$\Delta G_{\sigma }=\Delta G_{\sigma }\left(\sigma_{e},\sum ,\theta \right),$$

where $\sum =\frac{p}{\sigma_{e}}$, $p=\frac{\sigma_{kk}}{3}$ is the hydrostatic stress, $\theta =\frac{1}{3}\arcsin\left(-\frac{27}{2}\frac{\det s}{\sigma_{e}^{3}}\right)$, s=σ−pδ is the stress deviator, and δ is the second-order identity tensor.

Based on the small-particle experiments of Cech and Turnbull [62] in Fe–30%Ni alloys, Cohen and Olson [63] derived the following expression for the cumulative defect potency distribution per unit austenite volume $N_{v}$$\left(m^{-3}\right)$:

(8) $$N_{v}\left(T,X,\sigma \right)=N_{v}^{0}\;\exp\left(-a\;n^{*}\right)=N_{v}^{0}\;\exp\left(\frac{2\;a\;\gamma_{s}/\rho }{\Delta G_{ch}+\Delta G_{\sigma }+E_{str}+W_{f}}\right),$$

where (6) was used, $N_{v}^{0}$$\left(m^{-3}\right)$ is the total number of all potential nucleation sites per unit austenite volume and *a* is a shape factor constant. $N_{v}$ defines the number of nucleation sites per unit austenite volume with sufficient potency to nucleate martensite (operational sites).

A uniform distribution of austenite particles with average volume $v_{p}$ is considered. It is assumed that a single nucleation event transforms the particle into martensite. Therefore, the fraction *f* of particles to transform through sites of cumulative number density $N_{v}$ corresponds to the probability of finding at least one nucleation site in the particle (Cohen and Olson [63]):

(9) $$\begin{array}{lll} f(T,X,\sigma ) & = & c^{\left(a\right)}\left(1-\exp\left(-v_{p}\;N_{v}\right)\right]=\\ & = & c^{\left(a\right)}\left\{1-\exp\left[-v_{p}\;N_{v}^{0}\;\exp\left(\frac{2\;a\;\gamma_{s}/\rho }{\Delta G_{ch}+\Delta G_{\sigma }+E_{str}+W_{f}}\right)\right]\right\}, \end{array}$$

where (8) was used and $c^{\left(a\right)}$ is the available volume fraction of retained austenite for transformation.

For given temperature *T*, composition *X*, and average particle size $v_{p}$, Equation (9) defines *f* in terms of stress σ. Equation (9) can be described by the equivalent form

(10) $$\ln\left[-\frac{\ln\left(1-\frac{f}{c^{\left(a\right)}}\right)}{v_{p}\;N_{v}^{0}}\right]=\frac{2\;a\;\gamma_{s}/\rho }{\Delta G_{ch}\left(T,X\right)+\Delta G_{\sigma }\left(\sigma_{e},\sum ,\theta \right)+E_{str}+W_{f}\left(X\right)}.$$

For given triaxiality ∑ and Lode angle θ, last equation defines implicitly $\sigma_{e}$ in terms of temperature *T*, chemical composition *X*, average particle size $v_{p}$, and martensite volume fraction *f*, i.e., it yields a relationship of the form

(11) $$\sigma_{e}=\sigma_{e}\left(T,X,v_{p},\frac{f}{c^{\left(a\right)}},\sum ,\theta \right).$$

The temperature $M_{s}^{\sigma }$ is defined as the maximum temperature at which transformation is induced by a stress below the yield stress of the parent phase (Bolling and Richman [64], Olson and Cohen [65]). For temperatures below $M_{s}^{\sigma }$, an elastic stress-assisted transformation takes place at the same pre-existing nucleation sites, which are responsible for the spontaneous transformation on cooling. Let $\sigma_{y}\left(T\right)$ be the temperature-dependent yield stress of austenite. The experimental data of Olson and Azrin [1] show clearly that for $T\leq M_{s}^{\sigma }$, the 1% martensite curves are almost coincident with the 0.2% yield stress curves (Figures 5 and 6 in [1]), i.e., $\sigma =\sigma_{y}$ and $f/c^{\left(a\right)}\equiv f_{1}=0.01$ at $T=M_{s}^{\sigma }$. Therefore, by balancing the transformation stress in (11) with the $\sigma_{y}\left(T\right)$, the $M_{s}^{\sigma }$ temperature can be obtained, i.e., $M_{s}^{\sigma }$ is defined from the condition

(12) $$\sigma_{e}\left(M_{s}^{\sigma },X,v_{p},f_{1},\sum ,\theta \right)=\sigma_{y}\left(M_{s}^{\sigma }\right).$$

Equation (10) leads to the following solution for $M_{s}^{\sigma }$:

(13) $$\ln\left[-\frac{\ln(1-f_{1})}{v_{p}\;N_{v}^{0}}\right]=\frac{2\;a\;\gamma_{s}/\rho }{\Delta G_{ch}\left(M_{s}^{\sigma },X\right)+\Delta G_{\sigma }\left(\sigma_{y}\left(M_{s}^{\sigma }\right),\sum ,\theta \right)+E_{str}+W_{f}\left(X\right)}.$$

Last equation defines implicitly the $M_{s}^{\sigma }$ temperature in terms of chemical composition *X*, average particle size $v_{p}$, and the stress state parameters (∑,θ):

(14) $$M_{s}^{\sigma }=M_{s}^{\sigma }\left(X,v_{p},\sum ,\theta \right).$$

A detailed calculation of $M_{s}^{\sigma }$ for the TRIP steel under consideration is developed next. Haidemenopoulos et al. [66], based on the data of Olson and Cohen [65], suggest the following expression for $\Delta G_{\sigma }$, which depends on $\sigma_{e}$ and ∑, but is independent of θ:

(15) $$\Delta G_{\sigma }\left(\sigma_{e},\sum \right)=-\left(0.715\;\sigma_{e}+0.3206\;p\right)=-\sigma_{e}\left(0.715+0.3206\;\sum \right)\;\frac{J}{\mathrm{mol}}\;\left(p,\sigma_{e}\;\;\mathrm{in}\mathrm{MPa}\right).$$

The individual terms of the chemical driving force $\Delta G_{ch}$ and the frictional work of interfacial motion $W_{f}$ in Equation (6) were discussed in detail in the work of Haidemenopoulos and Vasilakos [15], who propose the following forms

(16) $$\begin{array}{l} \;\;\;\;\;\;\;\;\;\;\Delta G_{ch}\left(T,X_{C},X_{Mn}\right)=-[7381.6-69447\;X_{C}-19296\;X_{Mn}+38776\;X_{C}\;X_{Mn}\\ \;\;\;\;\;\;\;\;\;\;\;-\left(6.7821-33.45\;X_{C}\right)T]\;\frac{J}{\mathrm{mol}}, \end{array}$$

(17) $$W_{f}\left(X_{C},X_{Mn}\right)=1169+8777\;X_{C}+2246\;X_{Mn}+19900\;X_{C}\;X_{Mn}\;\frac{J}{\mathrm{mol}},$$

where $X_{C},X_{Mn}$ are the mole fractions of C and Mn in the retained austenite respectively, and *T* is the temperature in K. According to Olson and Cohen [58], the elastic strain energy is $E_{str}=500$ J/mol.

The yield stress data of Samek et al. [16] for the austenite present in TRIP steels show that the austenite flow stress decreases with temperature at a rate of 1 MPa/°C. Therefore, $\sigma_{y}\left(T\right)$ is described by

(18) $$\sigma_{y}\left(T\right)=\sigma_{0}-B\left(T-T_{0}\right),$$

where $\sigma_{0}=530$ MPa is the austenite flow stress at a temperature $T_{0}=25\;{}^{\circ}C=298.15$ K and B=1 MPa/K.

Substitution of (15)–(18) in (13) leads to the following solution for $M_{s}^{\sigma }$:

(19) $$M_{s}^{\sigma }=\frac{A+5712.6-78224\;X_{C}-21542\;X_{Mn}+18876\;X_{C}\;X_{Mn}+\left(0.715+0.3206\;\sum \right)\left(\sigma_{0}+B\;T_{0}\right)}{6.7821-33.45\;X_{C}+\left(0.715+0.3206\;\sum \right)B},$$

with

(20) $$A=\frac{\frac{2\;a\;\gamma_{s}}{\rho }}{\ln\left[-\frac{\ln(1-f_{1})}{v_{p}\;N_{v}^{0}}\right]},$$

where *A* is determined from (20) in J/mol, $\sigma_{0}$ is in MPa, *B* in MPa/K, $T_{0}$ in K, and $M_{s}^{\sigma }$ is calculated from (19) in K.

### 4.2. Calculation of $M_{s}^{\sigma }$ and the Effects of the Variants on Austenite Stability

Based on the small-particle experiments of Cech and Turnbull [62] in Fe–30%Ni alloys, Cohen and Olson [63] evaluated the shape factor *a* in (8) to be α=0.84. The size of the particles involved in those studies was of the order of 10 μm. In the TRIP steel under consideration the austenite particles are dispersed and their size is smaller than 1 μm. Therefore, the shape factor takes the smaller value a=0.12 (Haidemenopoulos et al. [17]). The values of the remaining parameters in Equation (20) are as follows [15,55]: $\gamma_{s}=0.15$$J/m^{2}$, $\rho =3\times 10^{-5}$$\mathrm{mol}/m^{2}$, and $N_{v}^{0}=2\times 10^{17}$ $m^{-3}$. Under the assumption that the austenite particles are spheroidal, the austenite particle volume $v_{p}=\frac{4}{3}\;\pi \;R^{3}$ was calculated using the mean austenite particle radius R=0.8 μm, as determined in the phase field calculations of Section 2.3. The yield strength of retained austenite is equal to $\sigma_{0}=530$ MPa.

The calculation of the $M_{s}^{\sigma }$ temperature is carried out in two steps. First, the effects of various parameters on the retained austenite stability are thoroughly examined. Then, the $M_{s}^{\sigma }$ temperature is calculated using (19) together with (20).

**Figure 7:**
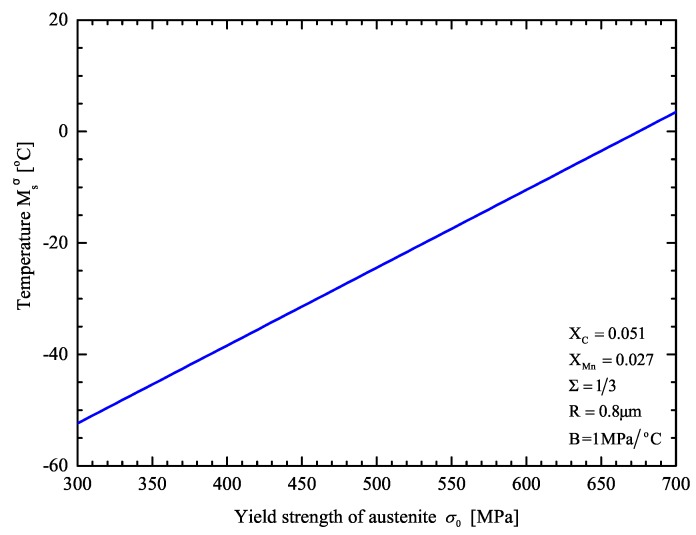
$M_{s}^{\sigma }$ temperature as a function of yield strength $\sigma_{0}$ of retained austenite.

For the case of uniaxial tension (∑=1/3), $M_{s}^{\sigma }$ is plotted as a function of the yield strength of austenite $\sigma_{0}$ in Figure 7.

An increase in the yield strength leads to an increase in the $M_{s}^{\sigma }$ temperature.

**Figure 8:**
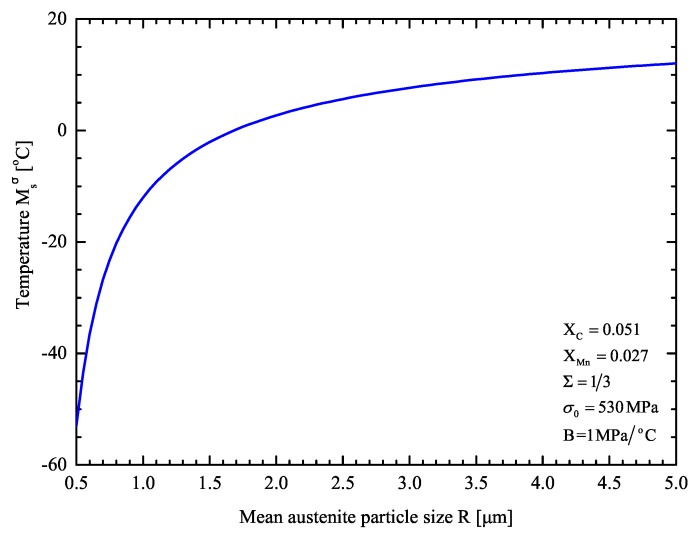
$M_{s}^{\sigma }$ temperature as a function of mean austenite particle in retained austenite.

Figure 8 shows the calculated $M_{s}^{\sigma }$ temperature with respect to the mean austenite size in uniaxial tension. The effect of particle size refinement on the austenite stability is evident: $M_{s}^{\sigma }$ decreases as the austenite dispersion becomes more refined. The predictions of the model are in agreement with the multitechnique investigation of Haidemenopoulos et al. [67].

**Figure 9:**
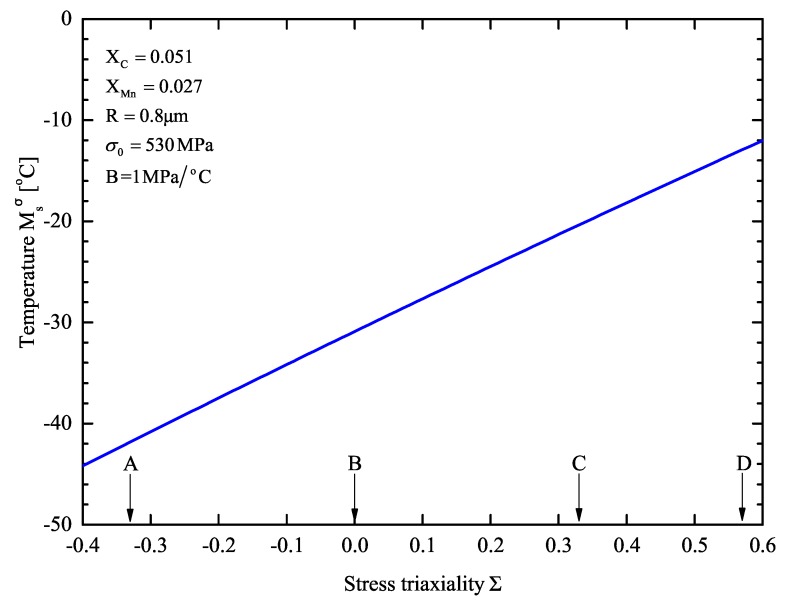
$M_{s}^{\sigma }$ temperature as a function of stress triaxiality $\left(\sum =p/\sigma_{e}\right)$ in retained austenite. (A): Uniaxial compression (∑=−1/3), (B): Pure shear (∑=0), (C): Uniaxial tension (∑=1/3), (D): Plane strain tension $\left(\sum =1/\sqrt{3}=0.577\right)$.

The effect of stress state on $M_{s}^{\sigma }$ is shown in Figure 9 for R=0.8μm. Four cases regarding the stress triaxiality are marked on the triaxiality axis: ∑ equals −1/3 in uniaxial compression, 0 in pure shear, 1/3 for tension, and $1/\sqrt{3}=0.5777$ in plane strain tension. The destabilizing effect of stress triaxiality is evident: the higher the value of stress triaxiality ∑, the higher the $M_{s}^{\sigma }$. Between the two extreme cases A (uniaxial compression) and D (plane strain tension) a 30 °C rise in $M_{s}^{\sigma }$ is observed.

For the TRIP steel under consideration the $M_{s}^{\sigma }$ temperature in uniaxial tension is predicted to be −20.3°C. This is in reasonable agreement with the measured value of −19 °C with the SS-TV-TT technique, as reported in Section 3.2.

### 4.3. Transformation Kinetics of Retained Austenite

The model developed by Haidemenopoulos et al. [17], for the description of the strain-induced martensitic transformation kinetics of dispersed austenite, is calibrated using available experimental data for the evolution of martensite volume fraction *f* in a uniaxial tension test for the TRIP700 steel under consideration (Bellas [68], Haidemenopoulos et al. [67]).

Kuroda [69] suggested that, in strain-induced transformation, the potency distribution $N_{v}$ in Equation (8) should depend on both stress and strain, so that $N_{v}=N_{v}^{\sigma }+N_{v}^{\varepsilon }$, where $N_{v}^{\sigma }$$\left(m^{-3}\right)$ is still defined by an equation similar to (8):

(21) $$N_{v}^{\sigma }\left(\sigma_{e},\sum \right)=N_{v}^{\sigma 0}\exp\left[-a_{\sigma }\;n^{*}\left(\sigma_{e},\sum \right)\right],$$

in which $N_{v}^{\sigma 0}$$\left(m^{-3}\right)$ denotes the pre-existing nucleation sites and $a_{\sigma }$ is a constant shape factor.

During plastic deformation, the austenite phase generates new more potent nucleation sites $N_{v}^{\varepsilon 0}$$\left(m^{-3}\right)$, which are calculated by an expression of the form (Haidemenopoulos et al. [17])

(22) $$N_{v}^{\varepsilon 0}\left({\bar{\varepsilon }}^{\left(a\right)}\right)=N\left\{1-\exp\left[-k{\left({\bar{\varepsilon }}^{\left(a\right)}\right)}^{m_{f}}\right]\right\},$$

where ${\bar{\varepsilon }}^{\left(a\right)}$ is the average equivalent plastic strain in austenite, *N*$\left(m^{-3}\right)$ is the maximum number of sites that can be produced by plastic strain, and $\left(k,m_{f}\right)$ are constants. As discussed in Haidemenopoulos et al. [17], the total number of operational sites due to the plastic strain in the austenite $N_{v}^{\varepsilon }$$\left(m^{-3}\right)$ is

(23) $$N_{v}^{\varepsilon }\left(\sigma_{e},\sum ,{\bar{\varepsilon }}^{\left(a\right)}\right)=N_{v}^{\varepsilon 0}\left({\bar{\varepsilon }}^{\left(a\right)}\right)\exp\left[-a_{\varepsilon }\;n^{*}\left(\sigma_{e},\sum \right)\right],$$

where $a_{\varepsilon }$ is the shape factor in the strain-modified potency distribution. Then, (9) takes the form [17]

(24) $$f\left(\sigma_{e},\sum ,{\bar{\varepsilon }}^{\left(a\right)}\right)=c^{\left(a\right)}\left[1-\exp\left(-v_{p}\;N_{v}\right)\right],\;N_{v}\left(\sigma_{e},\sum ,{\bar{\varepsilon }}^{\left(a\right)}\right)=N_{v}^{\sigma }\left(\sigma_{e},\sum \right)+N_{v}^{\varepsilon }\left(\sigma_{e},\sum ,{\bar{\varepsilon }}^{\left(a\right)}\right).$$

Equation (24) shows that the size of the austenite particles affects the amount of martensite produced during the transformation through the $v_{p}$ term.

#### Calibration of the Model to the Available Experimental Data

Equation (24) that describes the kinetics of strain-induced transformation was fitted non-linearly to available experimental data [67]. The maximum sites that can be formed by plastic deformation per unit volume *N* and the pre-existing nucleation sites $N_{v}^{\sigma 0}$ are the parameters to be determined.

The high chemical stability of the retained austenite (Figure 2d) prevents stress assisted transformation, i.e., the high value of $\Delta G_{ch}$ prevents the vanishing of $\gamma_{f}\left(n\right)$. In the TRIP steel under consideration, only the strain-induced transformation of retained austenite to martensite takes place. Therefore, the only parameter to be determined is *N* and the resulting value is $2.45\times 10^{22}$$m^{-3}$. The predicted evolution of *f* curve in uniaxial tension together with experimental data is presented in Section 6.

The values of the mean particle radius of the retained austenite, the chemical composition, the elastic strain energy $E_{str}$ and the rest of the parameters and constants are the same as those used in the calculation of $M_{s}^{\sigma }$ in Section 4.2. Since the experimental data of the volume fraction of martensite *f* were obtained at room temperature, the values of $\Delta G_{ch}=-1828$ J/mol and $W_{f}=1710$ J/mol were calculated at 25 °C using Equations (16) and (17) respectively.

The constants *k*, $m_{f}$, and $a_{\varepsilon }$ take the values [17]: $m_{f}=3.45$, k=46, and $a_{\varepsilon }=0.03$.

## 5. Description of the Constitutive Model

In this section, a constitutive model for TRIP steels is developed. A four-phase TRIP steel that consists of a ferritic matrix with a fine dispersion of bainite and austenite is considered; due to plastic deformation, the retained austenite transforms gradually into martensite. For each phase, the following labels are used: (1) for ferrite, (2) for bainite, (3) or (a) for austenite, and (4) or (m) for martensite. The constitutive equations are developed for finite strain problems.

The strain softening which results from the strain related to the transformation process, is an important characteristic of the martensitic transformation. An additional deformation rate is introduced into the constitutive model to take into account this strain softening. The total deformation rate can be written as the sum of an elastic, a plastic, and a transformation part:

(25) $$D=D^{e}+D^{p}+D^{TRIP}.$$

The elastic properties of all phases are essentially the same and standard isotropic linear hypoelasticity of homogeneous solids is used to define the elastic behavior of the composite material. The methodology presented by Papadioti et al. [26,70] is used to describe the plastic part $D^{p}$. The transformation part $D^{TRIP}$ has both deviatoric and volumetric parts and is in proportion to the rate of change of the volume fraction of martensite during martensitic transformation. Herein, the transformation kinetics model developed by Haidemenopoulos et al. [17] is used to describe the evolution of the volume fraction of martensite.

### 5.1. The Elastic Part $D^{e}$ of the Deformation Rate

The constitutive equation for $D^{e}$ is expressed as

(26) $$D^{e}=M^{e}:\overset{▽}{\sigma }\;\mathrm{or}\;\overset{▽}{\sigma }=L^{e}:D^{e},$$

where $\overset{▽}{\sigma }$ is the Jaumann derivative of the stress tensor σ, $M^{e}$ is the elastic compliance tensor defined as

(27) $$M^{e}=\frac{1}{2\;G}\;K+\frac{1}{3\;\kappa }\;J,\;L^{e}={M^{e}}^{-1}=2\;G\;K+3\;\kappa \;J,\;J=\frac{1}{3}\;\delta \;\delta ,\;K=I-J,$$

where *G* and κ stand for the elastic shear and bulk moduli, δ and I for the second- and symmetric fourth-order identity tensors, with Cartesian components $\delta_{ij}$ (the Kronecker delta) and $I_{ijkl}\;=\;\left(1/2\right)\left(\delta_{ik}\;\delta_{jl}+\delta_{il}\;\delta_{jk}\right)$.

### 5.2. Yield Criterion

The TRIP steel is treated as a rate-independent composite material with a yield function of the form (Papadioti et al. [26,70])

(28) $$\Phi \left(\sigma ,c^{\left(i\right)},{\bar{\varepsilon }}^{\left(i\right)}\right)=\sigma_{e}\left(\sigma \right)-{\tilde{\sigma }}_{0}\left(c^{\left(i\right)},{\bar{\varepsilon }}^{\left(i\right)}\right)=0,$$

where σ is the stress tensor, s=σ−pδ the stress deviator, $p=\sigma_{kk}/3$ the hydrostatic stress, and $\sigma_{e}$ the von Mises equivalent stress defined as

(29) $$\sigma_{e}=\sqrt{\frac{3}{2}\;s:s}\;,$$

$c^{\left(i\right)}$ (i=1,2,3,4) the volume fractions of the phases, and ${\bar{\varepsilon }}^{\left(i\right)}$ the average equivalent plastic strain in phase *i*. The effective yield stress ${\tilde{\sigma }}_{0}$ depends on the hardening behavior and the volume fractions of the constituent phases and is calculated from a constrained optimization problem:

(30) σ˜0(c(i),ε¯(i))=infy(i)≥0y(1)=1∑r=1Nc(r)σ0(r)2y(r)∑q=1Nc(q)3y(q)+2∑s=1Nc(s)y(s)3y(s)+2−1,

where N=4 is the number of phases, and $\sigma_{0}^{\left(i\right)}\left({\bar{\varepsilon }}^{\left(i\right)}\right)$ are the flow stresses of the individual phases.

The methodology developed by Kaufman et al. [71] and the CONMAX software (http://www.netlib.org/opt/conmax.f) are used to solve the constrained optimization problem in (30).

### 5.3. The Plastic Part $D^{p}$ of the Deformation Rate

The plastic part of the deformation rate $D^{p}$ is determined from “normality” to the yield surface:

(31) $$D^{p}=\dot{\bar{\varepsilon }}{}^{p}\frac{\partial \Phi }{\partial \sigma }=\dot{\bar{\varepsilon }}{}^{p}\;N,\;N=\frac{3}{2\;\sigma_{e}}\;s,\;\dot{\bar{\varepsilon }}{}^{p}=\sqrt{\frac{2}{3}\;D^{p}:D^{p}},$$

where $\bar{\varepsilon }{}^{p}$ is the non-negative equivalent plastic strain.

Homogenization theory also provides estimates for the average value of the deformation rate $D^{\left(i\right)}$ in the individual phases, which are written in the form (Papadioti et al. [26,70])

(32) $$D^{\left(i\right)}=\alpha^{\left(i\right)}\;D^{p},\;\alpha^{\left(i\right)}=\frac{{\hat{y}}^{\left(i\right)}}{3\;{\hat{y}}^{\left(i\right)}+2}{\left(\sum_{s=1}^{N}\frac{c^{\left(s\right)}\;{\hat{y}}^{\left(s\right)}}{3\;{\hat{y}}^{\left(s\right)}+2}\right)}^{-1}\;,$$

where ${\hat{y}}^{\left(r\right)}$ are the optimal values of $y^{\left(r\right)}$ calculated from the optimization problem in (30).

The average equivalent plastic strain rate in the phases ${\dot{\bar{\varepsilon }}}^{\left(i\right)}$ is defined as

$${\dot{\bar{\varepsilon }}}^{\left(i\right)}=\sqrt{\frac{2}{3}\;D^{\left(i\right)}:D^{\left(i\right)}}\;.$$

Then, (32a) yields

(33) $${\dot{\bar{\varepsilon }}}^{\left(i\right)}=\alpha^{\left(i\right)}\;\dot{\bar{\varepsilon }}{}^{p}.$$

The average equivalent plastic strain in the phases ${\bar{\varepsilon }}^{\left(i\right)}$, which is calculated from the time integration of ${\dot{\bar{\varepsilon }}}^{\left(i\right)}$, is used to describe the hardening of the phases. In particular, the flow stress $\sigma_{0}^{\left(i\right)}$ of each phase is assumed to be a function of the corresponding average equivalent plastic strain in the phase, i.e., $\sigma_{0}^{\left(i\right)}=\sigma_{0}^{\left(i\right)}\left({\bar{\varepsilon }}^{\left(i\right)}\right)$.

### 5.4. The Transformation Part $D^{TRIP}$ of the Deformation Rate

Finally, the transformation part $D^{TRIP}$ is defined as (Stringfellow et al. [18]):

(34) $$D^{TRIP}=\dot{f}\left[A\left(\sigma_{e}\right)\;N+\frac{1}{3}\;\Delta_{v}\;\delta \right],\;\mathrm{where}\;N=\frac{3}{2\;\sigma_{e}}\;s,\;A\left(\sigma_{e}\right)=A_{0}+A_{1}\frac{\sigma_{e}}{s_{a}^{*}},$$

$f=c^{\left(4\right)}$ denotes the volume fraction of martensite, a superposed dot stands for the material time derivative, $A_{0}$, $A_{1}$ and $\Delta_{v}$ are dimensionless parameters, and $s_{a}^{*}$ is a reference stress. In (34a), the first term is deviatoric and accounts for “shape changes” caused by martensitic transformation, and the second term is volumetric $\left(D_{kk}^{TRIP}=\dot{f}\;\Delta_{v}\right)$.

### 5.5. The Total Inelastic Deformation Rate $D^{in}\equiv D^{p}+D^{TRIP}$

The inelastic deformation rate is defined as the total of the plastic and transformation parts:

(35) $$D^{in}\equiv D^{p}+D^{TRIP},$$

so that

$$D=D^{e}+D^{in}.$$

Use of the constitutive equations for $D^{p}$ and $D^{TRIP}$ leads to

(36) $$D^{in}=\left(\dot{\bar{\varepsilon }}{}^{p}+\frac{A}{\Delta_{v}}{\dot{\varepsilon }}_{v}^{p}\right)N+\frac{1}{3}{\dot{\varepsilon }}_{v}^{p}\;\delta ,\;\mathrm{where}\;{\dot{\varepsilon }}_{v}^{p}=D_{kk}^{in}=\Delta_{v}\;\dot{f}.$$

### 5.6. Evolution of the Volume Fraction of the Phases

According to the transformation kinetics model presented in Section 4.3, the evolution equation for the volume fraction of martensite can be expressed as

(37) $$\dot{f}={\dot{c}}^{\left(4\right)}=c^{\left(a\right)}\;A_{f}\;{\dot{\bar{\varepsilon }}}^{\left(a\right)}\equiv g^{\left(4\right)}\;{\dot{\varepsilon }}_{v}^{p},\;\mathrm{or}\;{\dot{\varepsilon }}_{v}^{p}=\Delta_{v}\;\dot{f}=\Delta_{v}\;c^{\left(a\right)}\;A_{f}\;{\dot{\bar{\varepsilon }}}^{\left(a\right)},$$

where $c^{\left(a\right)}=c^{\left(3\right)}$ denotes the volume fraction of austenite, ${\bar{\varepsilon }}^{\left(a\right)}={\bar{\varepsilon }}^{\left(3\right)}$ is the average equivalent plastic strain in the austenite, and $A_{f}$ is determined by Haidemenopoulos et al. [17]

(38) $$A_{f}\left({\bar{\varepsilon }}^{\left(a\right)},\sum \right)=v_{p}\;a\left[N-N_{v}^{\varepsilon 0}\left({\bar{\varepsilon }}^{\left(a\right)}\right)\right]{\left({\bar{\varepsilon }}^{\left(a\right)}\right)}^{m_{f}-1}\;\exp\left[-a_{\varepsilon }\;n^{*}\left(\sum \right)\right],$$

where $N_{v}^{\varepsilon 0}$ is determined in Equation (23).

Next, the evolution equations for $c^{\left(1\right)}$, $c^{\left(2\right)}$, and $c^{\left(a\right)}$ are derived. Use of the definition $c^{\left(1\right)}=V^{\left(1\right)}/V$ leads to ${\dot{c}}^{\left(1\right)}=-c^{\left(1\right)}\;\dot{V}/V$. The contribution of elastic deformations to local volume change is ignored, so that $\dot{V}/V={\dot{\varepsilon }}_{v}^{p}=\Delta_{v}\;\dot{f}$ and

(39) $${\dot{c}}^{\left(1\right)}=-c^{\left(1\right)}\;{\dot{\varepsilon }}_{v}^{p}\equiv g^{\left(1\right)}\;{\dot{\varepsilon }}_{v}^{p}.$$

Likewise

(40) $${\dot{c}}^{\left(2\right)}=-c^{\left(2\right)}\;{\dot{\varepsilon }}_{v}^{p}\equiv g^{\left(2\right)}\;{\dot{\varepsilon }}_{v}^{p},$$

and, since $c^{\left(1\right)}+c^{\left(2\right)}+c^{\left(a\right)}+c^{\left(m\right)}=1$, ${\dot{c}}^{\left(a\right)}=-\left(\dot{f}+{\dot{c}}^{\left(1\right)}+{\dot{c}}^{\left(2\right)}\right)$ or

(41) $${\dot{c}}^{\left(a\right)}=-\left(\frac{1}{\Delta_{v}}-c^{\left(1\right)}-c^{\left(2\right)}\right)\;{\dot{\varepsilon }}_{v}^{p}\equiv g^{\left(3\right)}\;{\dot{\varepsilon }}_{v}^{p}.$$

### 5.7. The Elastoplastic Tangent Modulus

An equation relating the Jaumann derivative $\overset{\nabla }{\sigma }$ to the deformation rate D through the elastoplastic tangent modulus tensor L is derived from the elastoplastic constitutive equations. The derivation is as follows.

The elastic deformation rate $D^{e}$ is written as follows

$$D^{e}=D-D^{in}=D-\left[\left(\dot{\bar{\varepsilon }}{}^{p}+\frac{A}{\Delta_{v}}{\dot{\varepsilon }}_{v}^{p}\right)N+\frac{1}{3}{\dot{\varepsilon }}_{v}^{p}\;\delta \right].$$

Since ${\dot{\varepsilon }}_{v}^{p}=\Delta_{v}\;c^{\left(a\right)}\;A_{f}\;\alpha^{\left(a\right)}\;\dot{\bar{\varepsilon }}{}^{p}\equiv m\;\dot{\bar{\varepsilon }}{}^{p}$, last equation yields

$$D^{e}=D-\dot{\bar{\varepsilon }}{}^{p}\left[\left(1+m\;\frac{A}{\Delta_{v}}\right)N+\frac{m}{3}\;\delta \right].$$

Substitution of the above expression for $D^{e}$ into the hypoelastic constitutive Equation (26) $\left(\overset{\nabla }{\sigma }=L^{e}:D^{e}\right)$ results in

(42) $$\overset{\nabla }{\sigma }=L^{e}:\left\{D-\dot{\bar{\varepsilon }}{}^{p}\left[\left(1+m\;\frac{A}{\Delta_{v}}\right)N+\frac{m}{3}\;\delta \right]\right\}=L^{e}:D-\dot{\bar{\varepsilon }}{}^{p}\left[2\;G\;\left(1+m\;\frac{A}{\Delta_{v}}\right)N+m\;\kappa \;\delta \right],$$

where L:N=2GN and L:δ=3κδ were taken into consideration.

Φ is an isotropic function, therefore the “consistency condition” $\dot{\Phi }=0$ can be expressed as [72]

(43) $$\dot{\Phi }=\frac{\partial \Phi }{\partial \sigma }:\overset{\nabla }{\sigma }+\sum_{i=1}^{4}\left(\frac{\partial \Phi }{\partial {\bar{\varepsilon }}^{\left(i\right)}}\;{\dot{\bar{\varepsilon }}}^{\left(i\right)}+\frac{\partial \Phi }{\partial c^{\left(i\right)}}\;{\dot{c}}^{\left(i\right)}\right)=0.$$

Substitution of (42) for $\overset{\nabla }{\sigma }$ and of the evolution equations of ${\dot{\bar{\varepsilon }}}^{\left(i\right)}$ and ${\dot{c}}^{\left(i\right)}$ into the consistency condition leads to

(44) $$N:\left(L^{e}:D-\dot{\bar{\varepsilon }}{}^{p}\left[2\;G\;\left(1+\frac{A}{\Delta_{v}}\;m\;\right)N+m\;\kappa \;\delta \right]\right)+\sum_{i=1}^{4}\left(\frac{\partial \Phi }{\partial {\bar{\varepsilon }}^{\left(i\right)}}\;\alpha^{\left(i\right)}\;\dot{\bar{\varepsilon }}{}^{p}+\frac{\partial \Phi }{\partial c^{\left(i\right)}}\;g^{\left(i\right)}\;{\dot{\varepsilon }}_{v}^{p}\right)=0,$$

or

(45) $$2\;G\;N:D-3\;G\;\dot{\bar{\varepsilon }}{}^{p}\left(1+\frac{A}{\Delta_{v}}\;m\right)+\dot{\bar{\varepsilon }}{}^{p}\sum_{i=1}^{4}\left(\frac{\partial \Phi }{\partial {\bar{\varepsilon }}^{\left(i\right)}}\;\alpha^{\left(i\right)}+m\;\frac{\partial \Phi }{\partial c^{\left(i\right)}}\;g^{\left(i\right)}\right)=0,$$

where N:L=2GN, N:N=3/2, N:δ=0, and ${\dot{\varepsilon }}_{v}^{p}=m\;\dot{\bar{\varepsilon }}{}^{p}$ were taken into consideration. Last equation leads to

(46) $$\dot{\bar{\varepsilon }}{}^{p}=\frac{2\;G}{L}\;N:D,$$

where

(47) $$L=3\;G\;\left(1+m\;\frac{A}{\Delta_{v}}\right)+H,\;H=-\sum_{i=1}^{4}\left(\frac{\partial \Phi }{\partial {\bar{\varepsilon }}^{\left(i\right)}}\;\alpha^{\left(i\right)}+m\;\frac{\partial \Phi }{\partial c^{\left(i\right)}}\;g^{\left(i\right)}\right).$$

Substitution of the expression for $\dot{\bar{\varepsilon }}{}^{p}$ from (46) into (42) leads to

(48) $$\begin{array}{c} \overset{\nabla }{\sigma }=L:D \end{array}\;\mathrm{with}\;\begin{array}{c} L=L^{e}-\frac{2\;G}{L}\left[2\;G\;\left(1+m\;\frac{A}{\Delta_{v}}\right)N\;N+m\;\kappa \;\delta \;N\right] \end{array}\;,$$

where $m=\Delta_{v}\;A_{f}\;c^{\left(a\right)}\;\alpha^{\left(a\right)}$.

The numerical integration of the constitutive equations in the context of a finite element formulation is discussed in Appendix A and Appendix B.

## 6. Comparison of the Constitutive Model with Experimental Data

The flow stresses in the phases are written as follows

(49) $$\sigma_{y}^{\left(r\right)}=\sigma_{y}^{\left(r\right)}\left({\bar{\varepsilon }}^{\left(r\right)}\right),$$

where *r* denotes the number of the phase, $\sigma_{y}^{\left(r\right)}$ the yield stress, and ${\bar{\varepsilon }}^{\left(r\right)}$ the equivalent plastic strain.

The hardening behavior of the phases is determined through detailed literature search. The hardening behavior of ferrite, bainite, and martensite were acquired from experimental data presented in “Technical Steel Research” [73]. In particular, data for the annealed ferritic steel DOCOL 600 were used to determine the flow curve $\sigma_{y}^{\left(1\right)}$ of ferrite. To determine $\sigma_{y}^{\left(2\right)}$ for the bainitic phase, data from uniaxial tension tests conducted on a 0.5% C steel subjected to bainitic treatment (coiling temperature at 950 °C) are used. Data for the partially martensitic steel DOCOL 1400 with a high volume fraction (95%) of martensite are used to find $\sigma_{y}^{\left(m\right)}$. The resulting expressions at 25°C are

(50) $$\sigma_{y}^{\left(1\right)}=350{\left(1+\frac{{\bar{\varepsilon }}^{\left(1\right)}}{0.0042}\right)}^{\frac{1}{5.7}},\;\;\sigma_{y}^{\left(2\right)}=825{\left(1+\frac{{\bar{\varepsilon }}^{\left(2\right)}}{0.0104}\right)}^{\frac{1}{10.36}},\;\;\sigma_{y}^{\left(m\right)}=1132{\left(1+\frac{{\bar{\varepsilon }}^{\left(m\right)}}{0.0004}\right)}^{\frac{1}{16.65}},$$

where the flow stresses $\sigma_{y}^{\left(i\right)}$ are in MPa.

It appears that there are no reliable data for the flow curve of pure austenite in the literature. An estimation for $\sigma_{y}^{\left(a\right)}$ is given in Equation (51) below, based on the following reasoning. In the production of TRIP steels, the final step that follows intercritical annealing is isothermal holding in the bainite transformation range. During the formation of bainitic ferrite, carbon is rejected to the retained austenite, and the carbon content of the retained austenite is raised to values above 1 wt% [74]. This provides chemical stabilization and raises the austenite yield strength considerably; values in the range of 500–550 MPa were reported [16,75]. Therefore, the stress-strain curve of retained austenite is expected to lie above that of ferrite, which exhibits a lower yield strength due to its very low carbon content. The following expression is used for the flow curve austenite at 25°C:

(51) $$\sigma_{y}^{\left(a\right)}=530{\left(1+\frac{{\bar{\varepsilon }}^{\left(a\right)}}{0.08}\right)}^{\frac{1}{4.2}}\;\mathrm{in}\mathrm{MPa}.$$

**Figure 10:**
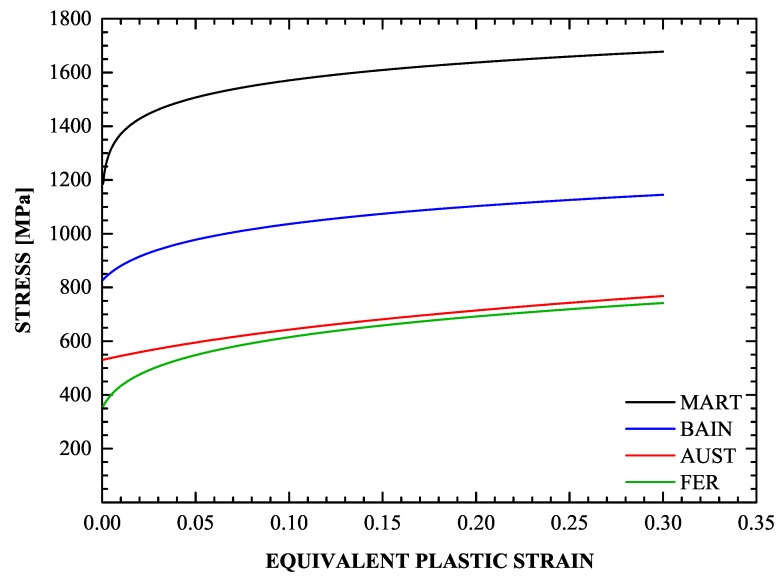
Hardening behavior of the constituent phases for the four-phase TRIP steel.

Figure 10 illustrates the hardening curves of the individual phases as given by (50) and (51).

### Experiments

**Figure 11:**
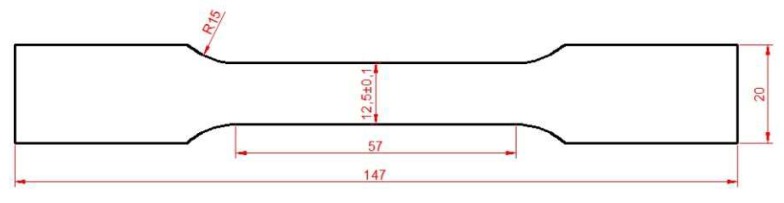
Geometry of tensile specimen (ASTM E8M).

The mechanical properties of the TRIP material were evaluated via uniaxial tension tests. An INSTRON 8801 servo-hydraulic machine with 100 kN load capacity was employed for the tensile tests. Mechanical properties were determined according to the ASTME8M at a constant crosshead velocity of 0.5 mm/min. Two specimens were tested in the longitudinal (L) direction using a 25-mm gage length clip-on extensometer. The geometry of the specimen used for the tensile tests is depicted in Figure 11.

The mechanical properties derived from the stress-strain curve are presented in Table 3. The yield stress and ultimate tensile strength is 530 MPa and 762 MPa, respectively. A high strain hardening rate at early stages of plastic straining is observed. The stable RA microstructure results in a gradual austenite transformation [76] and progressive hardening until necking at a uniform elongation of 25.4% [77,78].

According to the simulation results of the heat treatment presented in Section 2.3, the initial volume fractions for the individual phases are $c^{\left(1\right)}=0.507$, $c^{\left(2\right)}=0.327$, $c^{\left(a\right)}=0.166$, and $c^{\left(m\right)}=0.0$.

The constitutive model was used together with the ABAQUS general purpose finite element code to study the problem of uniaxial tension and the results are compared with the corresponding experimental data. One four-node isoparametric axisymmetric finite element (CAX4H in ABAQUS) is used to solve the uniaxial tension problem.

In the calculations, the Young’s modulus is E=220 GPa and the Poisson ratio is ν=0.3. The relative volume change related to the martensitic transformation $\Delta_{v}$, used in the constitutive equation for $D^{TRIP}$ (Equation (34)), is taken to be $\Delta_{v}=0.02$.

**Figure 12:**
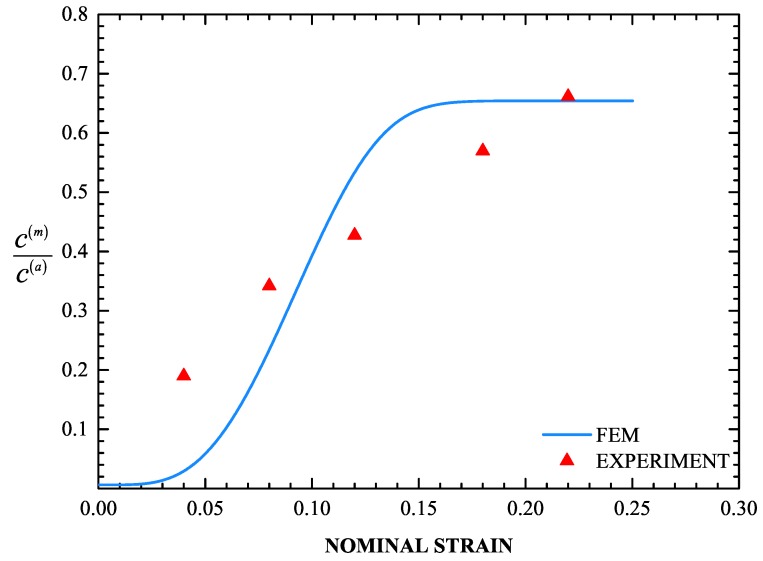
Calculated *f*-ε curve together with the experimental data (red triangles).

**Figure 13:**
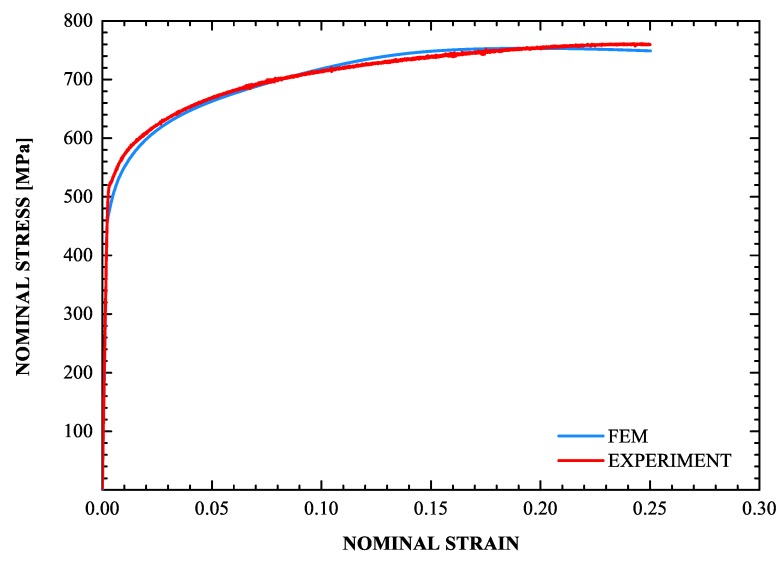
Calculated σ-ε curve (blue line) together with the experimental data (red line).

A very important part of the problem is the evolution of the volume fraction of martensite *f* during the uniaxial tension test. It is well known that the *f*-ε curve (ε= uniaxial strain) has a sigmoidal shape (Olson and Cohen [79]). The values of the parameters in the transformation kinetics model were defined in Section 4.3. Figure 12 and Figure 13 display the calculated *f*-ε and σ-ε curves and the corresponding experimental data, where σ is the nominal stress. The sigmoidal shape of the strain-induced transformation is predicted quite well by the transformation kinetics model. Initially, the transformation rate df/dε increases with strain, then reaches a rather constant rate and finally decreases at higher strains as saturation is achieved. Finally, for the temperature considered, the saturation level is lower than the value of 1, which corresponds to complete transformation. The model predictions agree well with the experimental data.

## 7. Applications

### 7.1. Necking of a Bar

**Figure 14:**
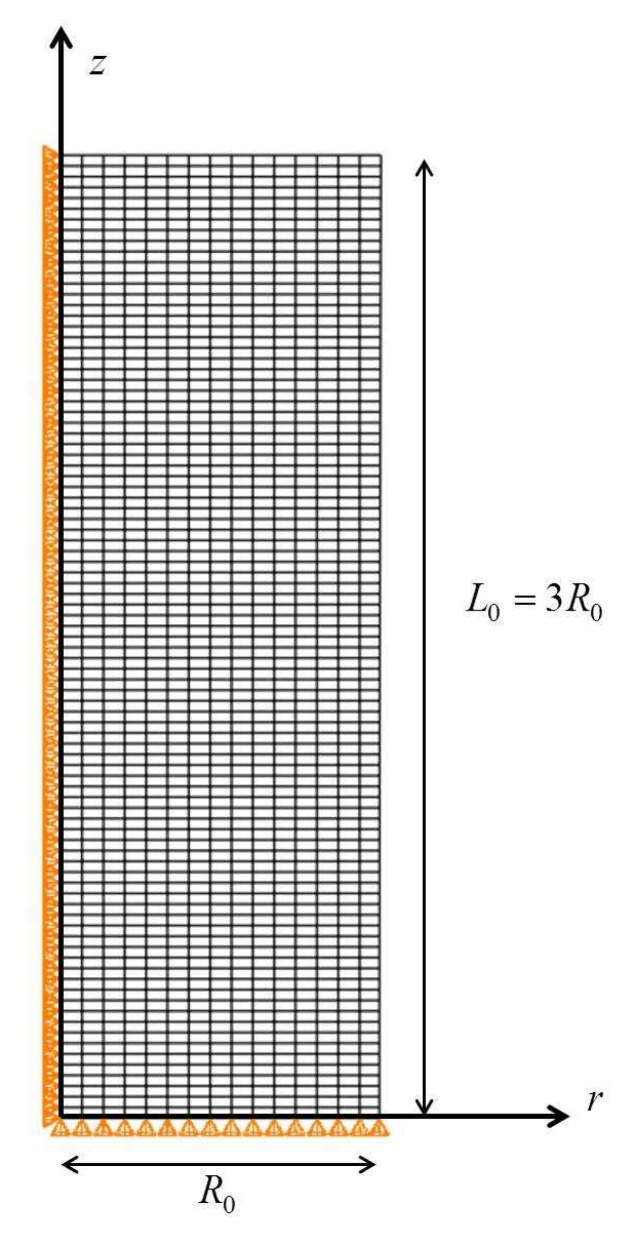
The finite element model employed for the analysis and a schematic representation of the boundary conditions imposed.

The constitutive model developed for the TRIP steel is used together with the finite element method to study the development of a neck in an axisymmetric tension specimen with a geometric imperfection. A cylindrical specimen with aspect ratio $L_{0}/R_{0}=3$ is considered, with $2\;L_{0}$ being the initial length and $R_{0}$ the initial radius. A cylindrical system is introduced as it is shown in Figure 14. Symmetric solutions are considered and only one half of the cylindrical specimen is studied. Figure 14 illustrates the finite element mesh used in the calculations; it comprises 1350 four-node isoparametric axisymmetric elements (CAX4H in ABAQUS) in a 15×90 grid. The following geometric imperfection is introduced to encourage necking:

(52) $$R\left(z\right)=R_{0}-\xi \;R_{0}\cos\frac{\pi \;z}{2\;L_{0}},$$

where R(z) is the perturbed radius and ξ is set equal to 0.005. Figure 14 shows the imposed geometric boundary conditions.

The deformation is driven by the uniform prescribed end displacement in the *z*-direction on the shear-free end $z=L_{0}$; the lateral surface on $r=R_{0}$ is kept traction free. The initial values of the volume fractions of the constituent phases are: $c_{0}^{\left(1\right)}=0.507$, $c_{0}^{\left(2\right)}=0.327$, $c_{0}^{\left(a\right)}=0.165$, and $c_{0}^{\left(m\right)}=0.001$. The flow curves $\sigma_{y}^{\left(r\right)}\;\left(r=1,4\right)$ of the phases are those presented in Section 6. Calculations are also performed for a non-transforming TRIP steel, in which the volume fractions of all phases are kept constant and equal to their initial values.

**Figure 15:**
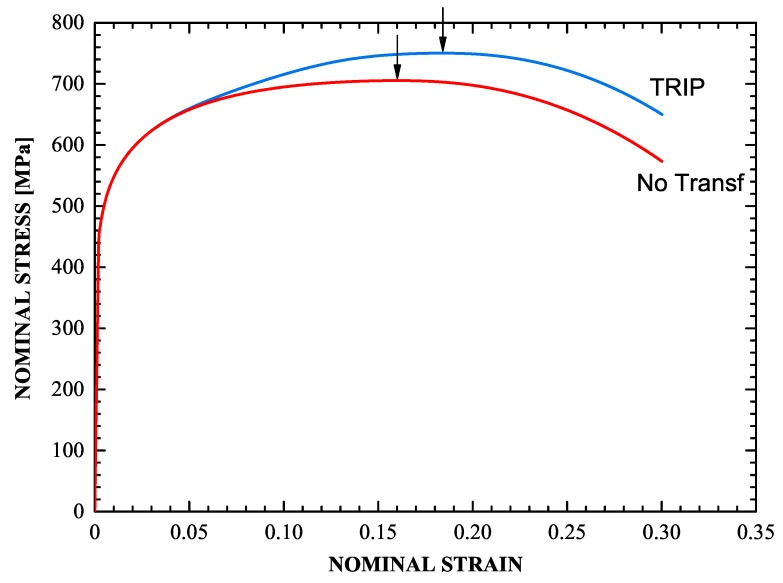
Stress-strain curves for a transforming steel and a “non-transforming” steel. The arrows show the position of the maximum load.

The uniaxial stress-strain curves of the imperfect specimen for both a transforming and a non-transforming material are depicted in Figure 15. The points of maximum load, which coincide with the end of uniform elongation in the corresponding specimen are denoted by the arrows. For the TRIP steel, the end of uniform elongation occurs at a nominal strain of 18.4% and 750 MPa stress, while for the non-transforming steel at 16% and 705 MPa respectively. Figure 15 shows that the TRIP phenomenon, in addition to strengthening the material, increases considerably the range of uniform elongation.

**Figure 16:**
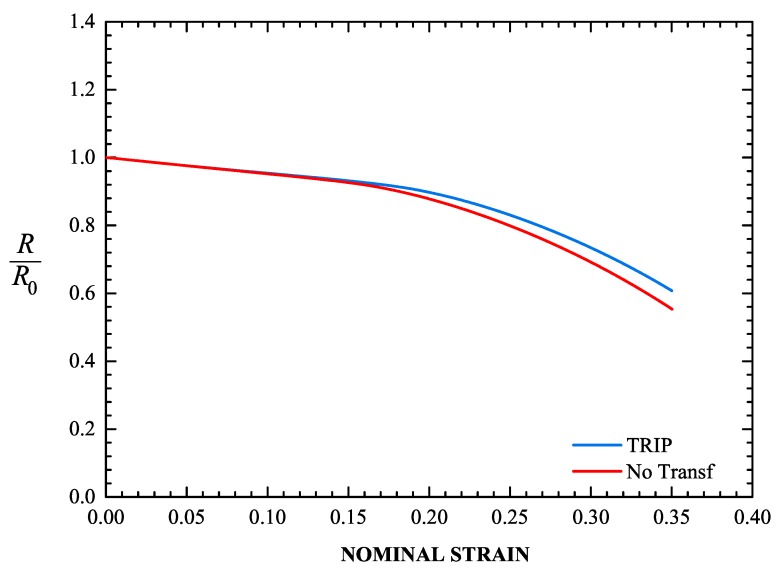
Evolution of the radius at the minimum cross section of the specimen for a transforming steel and a “non-transforming” steel.

**Figure 17:**
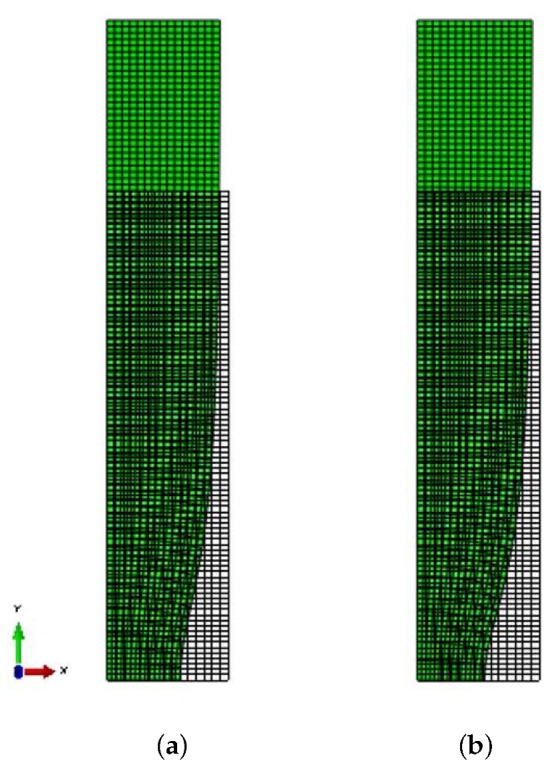
Deformed configurations for a nominal strain of 35%: (**a**) transforming, (**b**) “non-transforming” steel.

Figure 16 depicts the evolution of the radius at the minimum cross section of the specimen for the transforming and non-transforming materials, and Figure 17 shows the corresponding deformed configurations. At a nominal strain of 35%, the minimum cross section in the TRIP steel contracts 39.2%, whereas in the non-transforming material it contracts 44.7%. The formation of martensite stabilizes the neck and leads to its propagation down the length of the specimen. This result is consistent with the findings of Papatriantafillou et al. [19,80].

### 7.2. Forming Limit Diagrams

“Forming limit diagrams” for the TRIP steel under investigation are calculated using the constitutive model presented in the previous sections. Forming limit diagrams show the maximum deformation a sheet metal can be subjected to before the material fails by flow localization in a narrow straight band. Calculations are also carried out for a non-transforming steel with the same initial values of the volume fractions of the phases.

**Figure 18:**
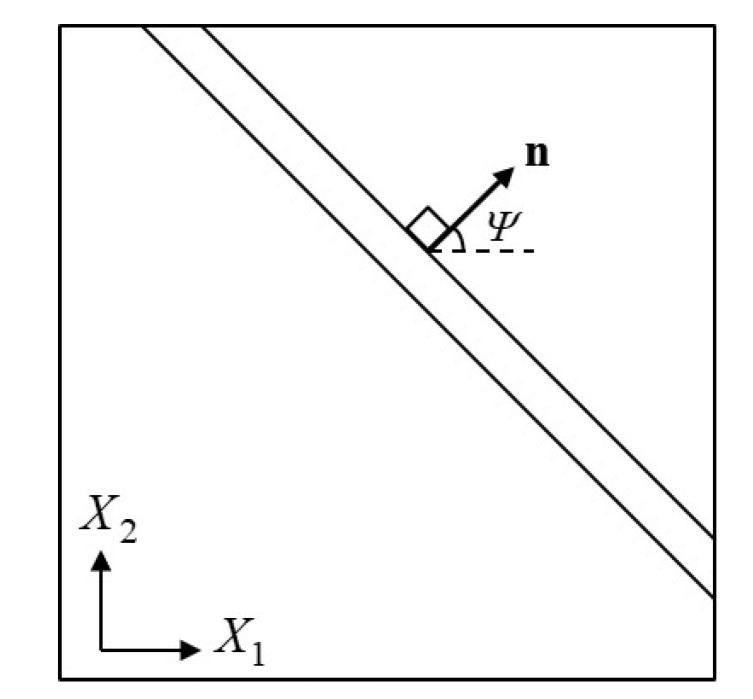
Narrow band in biaxially stretched sheet.

A sheet made of TRIP steel is deformed uniformly on its plane in a way that the in-plane principal strain increments increase proportionally. The possibility of the formation of an instability in the form of a narrow straight band (Figure 18) is studied.

As discussed in Section 5.7, the constitutive equations can be expressed as (Equation (48a))

(53) $$\overset{\nabla }{\sigma }=L:D,$$

where L is the elastoplastic tangent modulus tensor defined in (48b).

The formulation of the problem is more straightforward if “nominal” quantities are used. It can be readily shown that using the 1st Piola-Kirchhoff stress tensor $t=J\;F^{-1}\;\cdot \;\sigma$, the rate-constitutive Equation (53) can be written as

$$\dot{t}=R:{\dot{F}}^{T}\;or\;{\dot{t}}_{ij}=R_{ijkl}\;{\dot{F}}_{lk},$$

where J=detF,

$$R_{ijkl}=J\;F_{im}^{-1}\;F_{kn}^{-1}\left(L_{mjnl}+V_{mjnl}\right),\;\mathrm{and}\;V_{ijkl}=\frac{1}{2}\left(\sigma_{ik}\;\delta_{jl}-\delta_{ik}\;\sigma_{jl}-\sigma_{il}\;\delta_{jk}-\delta_{il}\;\sigma_{jk}\right)+\sigma_{ij}\;\delta_{kl}.$$

A state of uniform plane stress is assumed inside and outside the band and the possibility of the formation of a neck (band) as shown in Figure 18 is investigated. Let $X_{1}-X_{2}$ be the plane of the sheet and *H* the initial thickness of the sheet. Greek indices (α,β,γ,δ) are introduced and take values in the range (1,2). The in-plane displacements are continuous, therefore their spatial derivatives parallel to the band remain uniform. Let $\left[\partial u_{\alpha }/\partial X_{\beta }\right]$ be the displacement gradient discontinuities across the band, where [] denotes the difference of the field within the band and outside the band, $u_{\alpha }$ is the α-component (in-plane) of the displacement field, and $X_{\beta }$ the β-component of the position vector X of a material point in the *undeformed* configuration. It is a well known result in *Mechanics of Materials* that the only discontinuities in the displacement gradient are restricted kinematically to the following form (Hadamard [81], Hill [82], Rice [83])

(54) $$\left[\frac{\partial u_{\alpha }}{\partial X_{\beta }}\right]=G_{\alpha }\;N_{\beta },$$

where $N_{\beta }$ is the β-component of the unit vector N normal to the band in the *undeformed* configuration, and $G_{\alpha }$ the α-component of the vector G that defines the “jump” in the normal derivative of u, i.e., $\left[\partial u/\partial N\right]\equiv \left[\left(\partial u/\partial X\right)\;\cdot \;N\right]=G$. The vector G takes a constant value within the neck and depends on the imposed uniform deformation outside the neck. Next, a methodology for the calculation of G is presented.

Equation (54) leads to the conclusion that the in-plane components of the deformation gradient inside the band can be written as

$$F_{\alpha \beta }^{b}=F_{\alpha \beta }+G_{a}\;N_{\beta },$$

with superscript *b* denoting quantities inside the band, whereas quantities with no superscript refer to the uniform field outside the band. The deformation gradients in a matrix form are

(55) $$\left[F\right]=\left[\begin{array}{ccc} \lambda_{1} & 0 & 0\\ 0 & \lambda_{2} & 0\\ 0 & 0 & \lambda_{3} \end{array}\right]\;\mathrm{and}\;\left[F^{b}\right]=\left[\begin{array}{ccc} \lambda_{1}+G_{1}\;N_{1} & G_{1}\;N_{2} & 0\\ G_{2}\;N_{1} & \lambda_{2}+G_{2}\;N_{2} & 0\\ 0 & 0 & \lambda_{3}^{b} \end{array}\right],$$

where $\lambda_{i}$ are the stretch ratios. The plane stress condition $\sigma_{33}=0$ insinuates $t_{33}=0$. The condition ${\dot{t}}_{33}=0$ is solved for ${\dot{F}}_{33}$, so that the in-plane constitutive relations required for the sheet necking analysis are written in the form:

(56) $${\dot{t}}_{\alpha \beta }=C_{\alpha \beta \gamma \delta }\;{\dot{F}}_{\delta \gamma }\;\mathrm{with}\;C_{\alpha \beta \gamma \delta }=R_{\alpha \beta \gamma \delta }-R_{\alpha \beta 33}\frac{R_{33\gamma \delta }}{R_{3333}}.$$

Similarly, the in-plane constitutive relations within the band are

(57) $${\dot{t}}_{\alpha \beta }^{b}=C_{\alpha \beta \gamma \delta }^{b}\;{\dot{F}}_{\delta \gamma }^{b}=C_{\alpha \beta \gamma \delta }^{b}\;\left({\dot{F}}_{\delta \gamma }+{\dot{G}}_{\delta }\;N_{\gamma }\right)\;\mathrm{with}\;C_{\alpha \beta \gamma \delta }^{b}=R_{\alpha \beta \gamma \delta }^{b}-R_{\alpha \beta 33}^{b}\frac{R_{33\gamma \delta }^{b}}{R_{3333}^{b}}.$$

Equilibrium across the neck requires that

(58) $$T=T^{b},\;\mathrm{where}\;T_{\alpha }=H\;N_{\beta }\;t_{\beta \alpha }\;\mathrm{and}\;T_{\alpha }^{b}=H^{b}\;N_{\beta }\;t_{\beta \alpha }^{b}.$$

The rate form of this equilibrium relationship is

(59) $${\dot{T}}_{\alpha }\equiv H\;N_{\beta }\;{\dot{t}}_{\beta \alpha }=H^{b}\;N_{\beta }\;{\dot{t}}_{\beta \alpha }^{b}\equiv {\dot{T}}_{\alpha }^{b}.$$

Substitution of Equations (56) and (57) in (59) and use of Equation (55) for F and $F^{b}$ leads to the expression

(60) $$A\;\cdot \;\dot{G}=B\;\cdot \;\dot{b},$$

where

$$A_{\alpha \beta }=N_{\gamma }\;C_{\gamma \alpha \delta \beta }^{b}\;N_{\delta },\;B_{\alpha \beta }=N_{\gamma }\left(\frac{H}{H^{b}}\;C_{\gamma \alpha \beta \underline{\beta }}-C_{\gamma \alpha \beta \underline{\beta }}^{b}\right)\;\;\left(nosumon\beta \right),\;b_{\alpha }=\lambda_{\alpha }.$$

Equation (60) expresses the rate of equilibrium equation across the band, i.e., the rate of (58), in terms of $\dot{G}$. In (60), the jump G in the normal derivative of u across the band is determined in terms of the imposed uniform stretching through b$\left(b_{\alpha }=\lambda_{\alpha }\right)$.

In the case of a perfect sheet ($H^{b}=H$), the right hand side of (60) vanishes, since $H^{b}=H$ and $C^{b}=C$ lead to B=0, and the deformation continues to be homogenous ($\dot{G}=0$) until the condition for local necking bifurcation det[A]=0 is met.

The methodology of Marciniak and Kuzyski [84] is used in the calculations. A small initial imperfection is assumed to exist in the sheet and gradual localization of the strains at the imperfection leads to necking. In particular, a straight narrow band of reduced thickness $H^{b}<H$ is considered (Figure 18). A state of uniform plane stress inside and outside the band is assumed and the problem is to determine the uniform state of deformation within the band that is conforming kinematically and statically with the prescribed uniform state outside the band (Tvergaard [85,86], Needleman and Tvergaard [87]). When the sheet is not perfect $\left(H^{b}<H\right)$, the right hand side of (60) does not eliminate $\left(C^{b}\neq C\right)$, so (60) can be solved for $\dot{G}$. Since the initial sheet thickness inside and outside the band, and the imposed uniform deformation history F outside the band are known, Equations (60) are solved incrementally for $\Delta G=\dot{G}\;\Delta t$ to acquire the deformation history within the band. Localization takes place when the ratio of some scalar measure of the amount of incremental straining within the band to the corresponding value outside the band becomes unbounded.

The deformation gradient outside the band F is prescribed in a way that the principal logarithmic strains $\varepsilon_{1}$ and $\varepsilon_{2}$ outside the band increase proportionally:

$$\frac{d\varepsilon_{2}}{d\varepsilon_{1}}=\frac{\varepsilon_{2}}{\varepsilon_{1}}=\rho =\mathrm{const}.\;\mathrm{so}\;\mathrm{that}\;\lambda_{2}=\lambda_{1}^{\rho }.$$

The plane stress algorithm discussed in Appendix B is used to acquire the uniform solution outside the band. At the end of each increment, ΔG is calculated using Equation (60) and subsequently the deformation gradient within the band $F^{b}$ is defined. Next, the plane stress algorithm is used again to acquire the uniform solution within the band. The localization condition is met when $d|G|/d\lambda_{1}=\infty$, i.e., when det[A]=0 within the band.

To increase the precision of the calculations, instead of the rate of equilibrium Equation (60), equilibrium itself (58) is used:

(61) $$T_{n+1}=T_{n+1}^{b},$$

where the subscript n+1 denotes values at the end of the increment. Then, the approximations $T_{n+1}≃T_{n}+{\dot{T}}_{n}\;\Delta t=T_{n}+H\;N\;\cdot \;{\dot{t}}_{n}\;\Delta t$ and $T_{n+1}^{b}≃T_{n}^{b}+{\dot{T}}_{n}^{b}\;\Delta t=T_{n}+H\;N\;\cdot \;{\dot{t}}_{n}^{b}\;\Delta t$ are used in (61). Finally, (56) and (57) are used for ${\dot{t}}_{n}$ and ${\dot{t}}_{n}^{b}$, and (55) is used for F and $F^{b}$, to find

(62) $$A_{n}\;\cdot \;\Delta G=B_{n}\;\cdot \;\Delta b+\frac{1}{H^{b}}\left(T_{n}-T_{n}^{b}\right),$$

which is used for the determination of ΔG instead of (60). The last term on the right hand side of (62) takes into consideration any unbalanced forces at end of the previous increment.

The initial values of the volume fractions of the constituent phases are: $c_{0}^{\left(1\right)}=0.507$, $c_{0}^{\left(2\right)}=0.327$, $c_{0}^{\left(a\right)}=0.165$, and $c_{0}^{\left(m\right)}=0.001$. The curves $\sigma_{y}^{\left(r\right)},\;r=1,4$ that define the variation of the flow stress of the phases and the material data used in the calculations are those presented in Section 6. Calculations are also conducted for a non-transforming TRIP steel with the same initial values of the volume fractions of the phases.

The unit vector N can be expressed as $N=\cos\Psi \;e_{1}+\sin\Psi \;e_{2}$, with Ψ being the angle of inclination of the band relative to the $X_{1}$ axis in the undeformed configuration. For each value of $\rho =d\varepsilon_{2}/d\varepsilon_{1}$, calculations are conducted for values of Ψ covering the range 0°≤Ψ≤90° and determine the strain level at which the localization condition det[A]=0 is first met. The critical value $\Psi^{cr}$ for each ρ is defined as the one resulting in the minimum localization strain.

**Figure 19:**
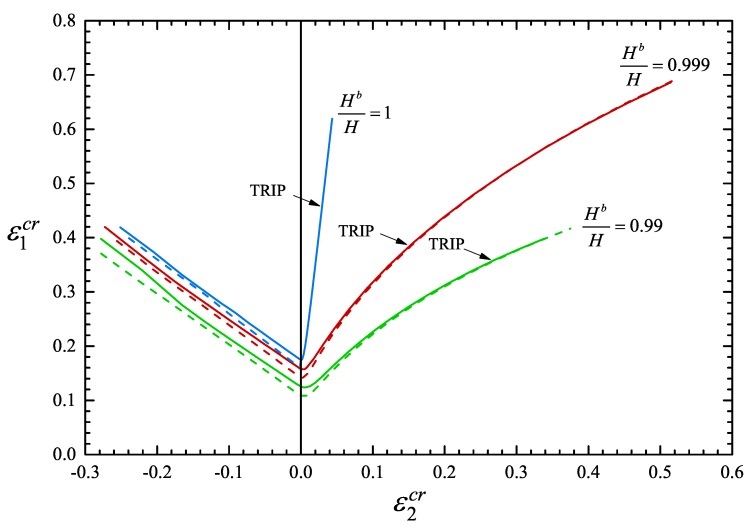
Forming limit curves for two different values of initial thickness inhomogeneities $H^{b}/H=0.999$ and $H^{b}/H=0.99$. The solid lines correspond to the TRIP steel, whereas the dashed lines are for a non-transforming steel.

Figure 19 depicts “forming limit curves” for proportional straining for a case without imperfection ($H^{b}/H=1$) and for two different values of the initial thickness imperfection, namely $H^{b}/H=0.999$ and $H^{b}/H=0.99$. The three solid curves denote the TRIP steel, while the dashed curves correspond to the non-transforming steel. It is observed that the TRIP phenomenon increases in general the necking localization strains. This result is consistent with the findings of Papatriantafillou et al. [19,80], who used a rate dependent constitutive model for TRIP steels (as opposed to the rate independent model used here). For the case of plane strain (ρ=0) and no imperfection, the critical strain $\varepsilon_{1}^{cr}$ increases from 0.1608 for the non-transforming steel to 0.1759 for the TRIP steel. The values of $\varepsilon_{1}^{cr}$ for $H^{b}/H=0.999$ and ρ=0 are 0.1417 for the non-transforming steel and 0.1575 for the TRIP steel; for $H^{b}/H=0.99$ and ρ=0, $\varepsilon_{1}^{cr}$ is 0.1083 for the non-transforming steel and 0.1254 for the TRIP steel.

## 8. Conclusions

The mechanical properties of TRIP steels are intrinsically associated with the stability of retained austenite, which results from the heat treatment design. In the current work, models for the heat treatment of TRIP steels, the austenite stability, the transformations kinetics of austenite as well as the mechanical behavior of the composite material were developed. The predictions of the models were then verified with an experimental study.

In particular, a 2D multi-phase field (MPF) model was employed for the prediction of the microstructural features of a CR-TRIP700 steel during a two-stage heat treatment, consisting of intercritical annealing, followed by an isothermal bainitic treatment. The MPF model is able to describe the temporal evolution of the volume fractions and the average grain size of the phases, as well as their average concentration in carbon, aluminum, and manganese. The phase-field results, obtained at the end of the heat treatment, were implemented in the $M_{s}^{\sigma }$ temperature model. Both the chemical enrichment and the size refinement of the retained austenite resulted in a rather stable retained austenite dispersion.

An experimental validation of the heat treatment process was performed. The measurements concerning the volume fraction and the average grain size of retained austenite were compared to the respective phase-field results. A good agreement between the experimental data and the phase-field simulation results was observed.

The $M_{s}^{\sigma }$ temperature model was employed to predict the stability of the retained austenite. In order to take advantage of the strain-induced transformation occurring in TRIP steels, $M_{s}^{\sigma }$ temperature should be bellow room temperature. The value of the $M_{s}^{\sigma }$ temperature predicted by the model is consistent with the experimental measurements of the SS-TV-TT technique. The yield strength, the mean particle size, and the stress state influence austenite stability. An increase in any of those parameters results in an increase in the $M_{s}^{\sigma }$ temperature, leading to a decrease in the stability of the retained austenite.

The phase-field results, obtained at the end of the heat treatment, were also implemented in a model for the transformation kinetics of retained austenite. The sigmoidal shape of the strain-induced transformation was predicted quite well by this model.

The transformations kinetics model and non linear homogenization techniques for non-linear composites were used to develop a constitutive model to describe the mechanical behavior of the TRIP steel under investigation. A method for the numerical integration of the constitutive model in the context of a finite element formulation was presented, and the model was introduced in a general-purpose finite element code (ABAQUS). In addition, a methodology for the numerical integration of the constitutive equations under plane stress conditions was discussed. One-element finite element calculations for the uniaxial tension problem were conducted, and the results were compared with experimental data obtained from uniaxial tension tests. The model predictions agree well with the experiments. The problem of necking under tension was analyzed thoroughly, and “forming limit diagrams” were calculated. In both cases it is evident that the TRIP phenomenon not only strengthens the material, but also increases considerably the range of uniform elongation.

The models developed provide an integrated simulation toolkit for the computer-assisted design of TRIP steels, which can be used to translate mechanical property requirements into optimised microstructural characteristics and to identify the appropriate processing routes. This methodology can be employed for other steel grades used in the automotive industry with the appropriate calibration.

## Figures and Tables

**Table 1:** Chemical composition of CR-TRIP700 in wt pct.

C	Mn	Si	Al
0.202	1.99	0.348	1.07

**Table 2:** Interfacial energy and interface mobility parameters of phase interactions.

	a−a	a−p	p−p	a−γ	γ−p	γ−γ
$\sigma_{ij}\;\;\left(J/m^{2}\right)$	0.5	0.9	1	0.4	0.5	0.7
$d_{ij}\;\;\left(Å\right)$	2.4	3.2	3.9	2.5	3.3	2.6
$Q_{ij}\;\;\left(\mathrm{kJ}/\mathrm{mol}\right)$	150	200	200	162.5	162.5	185

**Table 3:** Mechanical properties of TRIP steel.

	$\sigma_{y\;0.2}$ [MPa]	$\sigma_{\mathrm{UTS}}$ [MPa]	$A_{f}$ [%]	$A_{g}$ [%]	n	K [MPa]
TRIP700	530	762	28.6	25.14	0.166	1182

## Appendix A. Numerical Implementation of the Constitutive Model

In a finite element environment, the solution is developed incrementally, and the constitutive equations are integrated at the element Gauss integration points. At a Gauss point, the solution $\left(F_{n},\sigma_{n},c_{n}^{\left(r\right)},{\bar{\varepsilon }}_{n}^{\left(r\right)}\right)$ at time $t_{n}$ as well as the deformation gradient $F_{n+1}$ at time $t_{n+1}=t_{n}+\Delta t$ are known, and the problem is to determine $\left(\sigma_{n+1},c_{n+1}^{\left(r\right)},{\bar{\varepsilon }}_{n+1}^{\left(r\right)}\right)$.

Let $A_{n}$ and $A_{n+1}$ stand for the values of *A* at the start $t_{n}$ and at the end $t_{n+1}$ of the increment; the notation $\Delta A=A_{n+1}-A_{n}$ is used.

The effective flow stress ${\tilde{\sigma }}_{0}\left(c_{n+1}^{\left(i\right)},{\bar{\varepsilon }}_{n+1}^{\left(i\right)}\right)$ of the TRIP steel is assumed to remain constant over the time increment $\left(t_{n},t_{n+1}\right)$ and can be calculated from the optimization problem in (30), in which the flow stresses $\sigma_{0}^{\left(i\right)}$ of the phases take values (Papadioti et al. [26,70])

(A1) $$\sigma_{0}^{\left(i\right)}=\left(1-\beta \right)\sigma_{0}^{\left(i\right)}\left({\bar{\varepsilon }}_{n}^{\left(i\right)}\right)+\beta \;\sigma_{0}^{\left(i\right)}\left({\bar{\varepsilon }}_{n+1}^{\left(i\right)}\right),\;0\leq \beta \leq 1.$$

The optimal values of ${\hat{y}}_{n+1}^{\left(i\right)}$, which are used to define the strain concentration factors $\alpha^{\left(i\right)}$ in (32b) for the increment, are also calculated from the optimization problem (30). The calculation is implicit in general, except when β=0 is used in (A1).

In the following, an algorithm suitable for three-dimensional, plane strain, and axisymmetric problems is presented.

The constitutive equations are written as [19,80]

(A2) $$D=D^{e}+D^{in}\;\;\;\;\Rightarrow \;\;\dot{E}={\dot{E}}^{e}+{\dot{E}}^{in},$$

(A3) $$\overset{▽}{\sigma }=L^{e}:D^{e}\;\;\;\;\Rightarrow \;\;\dot{\hat{\sigma }}=L^{e}:{\dot{E}}^{e},$$

(A4) $$D^{in}=\left(\dot{\bar{\varepsilon }}{}^{p}+\frac{A}{\Delta_{v}}\;{\dot{\varepsilon }}_{v}^{p}\right)N+\frac{1}{3}\;{\dot{\varepsilon }}_{v}^{p}\;\delta \;\;\Rightarrow \;\;{\dot{E}}^{in}=\left(\dot{\bar{\varepsilon }}{}^{p}+\frac{A}{\Delta_{v}}\;{\dot{\varepsilon }}_{v}^{p}\right)\hat{N}+\frac{1}{3}\;{\dot{\varepsilon }}_{v}^{p}\;\delta ,$$

(A5) $$D^{\left(i\right)}=\alpha^{\left(i\right)}\;D^{p}\;\;\;\;\Rightarrow \;\;{\dot{\bar{\varepsilon }}}^{\left(i\right)}=\alpha^{\left(i\right)}\;\dot{\bar{\varepsilon }}{}^{p},$$

(A6) $$\Phi (\sigma ,c^{\left(i\right)},{\bar{\varepsilon }}^{\left(i\right)})=0\;\;\;\;\Rightarrow \;\;\sigma_{e}\left(\hat{\sigma }\right)-{\tilde{\sigma }}_{0}\left(c^{\left(i\right)},{\bar{\varepsilon }}^{\left(i\right)}\right)=0,$$

where the assumption was made that the Lagrangian triad associated with $\Delta F\left(t\right)=F\left(t\right)\;\cdot \;F_{n}^{-1}=R\left(t\right)\;\cdot \;U\left(t\right)$ (polar decomposition) remains fixed over the time increment $\left(t_{n},t_{n+1}\right)$, E(t)=lnU(t) is the Lagrangian logarithmic strain relative to the start of the increment $\left(E_{n}=0\right)$, $\hat{\sigma }\left(t\right)=R^{T}\left(t\right)\;\cdot \;\sigma \left(t\right)\;\cdot \;R\left(t\right)$, and $\hat{N}\left(t\right)=R^{T}\left(t\right)\;\cdot \;N\left(t\right)\;\cdot \;R\left(t\right)$.

The evolution equations of the volume fractions of the phases are:

(A7) $${\dot{c}}^{\left(1\right)}=-c^{\left(1\right)}\;{\dot{\varepsilon }}_{v}^{p},$$

(A8) $${\dot{c}}^{\left(2\right)}=-c^{\left(2\right)}\;{\dot{\varepsilon }}_{v}^{p},$$

(A9) $$\dot{f}={\dot{c}}^{\left(m\right)}={\dot{c}}^{\left(4\right)}=\frac{{\dot{\varepsilon }}_{v}^{p}}{\Delta_{v}}=c^{\left(a\right)}\;A_{f}\;{\dot{\bar{\varepsilon }}}^{\left(a\right)}=c^{\left(a\right)}\;A_{f}\;\alpha^{\left(a\right)}\;\dot{\bar{\varepsilon }}{}^{p},$$

(A10) $${\dot{c}}^{\left(3\right)}={\dot{c}}^{\left(a\right)}=-\left({\dot{c}}^{\left(1\right)}+{\dot{c}}^{\left(2\right)}+{\dot{c}}^{\left(m\right)}\right).$$

Equations (A4) that determines the inelastic deformation rate ${\dot{E}}^{in}$, (A5) that define the ${\bar{\varepsilon }}^{\left(i\right)}$, and (A9) that defines the evolution of the volume fraction of martensite *f*, all require numerical integration. The remaining equations are integrated exactly:

(A11) $$\dot{E}={\dot{E}}^{e}+{\dot{E}}^{in}\;\Rightarrow \;\Delta E^{e}=\Delta E-\Delta E^{in},$$

(A12) $${\hat{\sigma }}_{n+1}=\sigma_{n}+L^{e}:\Delta E^{e}=\sigma_{n}+L^{e}:\left(\Delta E-\Delta E^{in}\right)={\hat{\sigma }}^{e}-L^{e}:\Delta E^{in},$$

(A13) $$c_{n+1}^{\left(1\right)}=c_{n}^{\left(1\right)}\exp\left(-\Delta \varepsilon_{v}^{p}\right),$$

(A14) $$c_{n+1}^{\left(2\right)}=c_{n}^{\left(2\right)}\exp\left(-\Delta \varepsilon_{v}^{p}\right),$$

(A15) $$f_{n+1}=f_{n}+\frac{\Delta \varepsilon_{v}}{\Delta_{v}},$$

(A16) $$c_{n+1}^{\left(a\right)}=1-\left(f_{n+1}+c_{n+1}^{\left(1\right)}+c_{n+1}^{\left(2\right)}\right),$$

where ${\hat{\sigma }}^{e}=\sigma_{n}+L^{e}:\Delta E$ is the (known) “elastic predictor”. The rest of the equations are

(A17) $$\sigma_{e}{|}_{n+1}-{\tilde{\sigma }}_{0}\left(c_{n+1}^{\left(i\right)},{\bar{\varepsilon }}_{n+1}^{\left(i\right)}\right)=0,$$

(A18) $${\dot{E}}^{in}=\left(\dot{\bar{\varepsilon }}{}^{p}+\frac{A}{\Delta_{v}}\;{\dot{\varepsilon }}_{v}^{p}\right)\hat{N}+\frac{1}{3}\;{\dot{\varepsilon }}_{v}^{p}\;\delta ,\;\hat{N}=\frac{3}{2\;\sigma_{e}}\;\hat{s}\;A=A_{0}+A_{1}\frac{\sigma_{e}}{s_{a}^{*}},$$

(A19) $${\dot{\bar{\varepsilon }}}^{\left(i\right)}=\alpha^{\left(i\right)}\;\dot{\bar{\varepsilon }}{}^{p},$$

(A20) $${\dot{\varepsilon }}_{v}^{p}=\Delta_{v}\;c^{\left(a\right)}\;A_{f}\;\alpha^{\left(a\right)}\;\dot{\bar{\varepsilon }}{}^{p}.$$

The *backward* Euler method is used for the numerical integration of the inelastic deformation rate (A18), and the *forward* Euler scheme is used for the numerical integration of (A19) and (A20). Use of a backward Euler scheme for the numerical integration of ${\dot{E}}^{in}$ is imperative in order to be able to use increments of reasonable size (Aravas and Ponte Castañeda [88]). Numerical integration of (A18)–(A20) yields

(A21) $$\Delta E^{in}=\left(\Delta \bar{\varepsilon }{}^{p}+\frac{A_{n+1}}{\Delta_{v}}\Delta \varepsilon_{v}^{p}\right){\hat{N}}_{n+1}+\frac{1}{3}\;\Delta \varepsilon_{v}^{p}\;\delta ,\;A_{n+1}=A_{0}+A_{1}\frac{\sigma_{e}{|}_{n+1}}{s_{a}^{*}},$$

(A22) $$\Delta {\bar{\varepsilon }}^{\left(i\right)}=\alpha_{n}^{\left(i\right)}\;\Delta \bar{\varepsilon }{}^{p},$$

(A23) $$\Delta \varepsilon_{v}^{p}=\Delta_{v}\;c_{n}^{\left(a\right)}\;A_{f}{|}_{n}\;\alpha_{n}^{\left(a\right)}\;\Delta \bar{\varepsilon }{}^{p}.$$

Substitution of $L^{e}=2\;\mu \;K+3\;\kappa \;J$ and of Equation (A21) for $\Delta E^{in}$ in the elasticity Equation (A12) leads to

(A24) $${\hat{\sigma }}_{n+1}={\hat{\sigma }}^{e}-2\;G\left(\Delta \bar{\varepsilon }{}^{p}+\frac{A_{n+1}}{\Delta_{v}}\Delta \varepsilon_{v}^{p}\right){\hat{N}}_{n+1}-\kappa \;\Delta \varepsilon_{v}^{p}\;\delta .$$

Calculation of the deviatoric part of last equation and use the definition of ${\hat{N}}_{n+1}$ leads to the conclusion that the stress deviator ${\hat{s}}_{n+1}$ is proportional to the deviatoric part of the elastic predictor ${\hat{s}}^{e}$:

(A25) $${\hat{s}}_{n+1}={\hat{s}}^{e}-2\;G\left(\Delta \bar{\varepsilon }{}^{p}+\frac{A_{n+1}}{\Delta_{v}}\Delta \varepsilon_{v}^{p}\right)\frac{3}{2\;\sigma_{e}{|}_{n+1}}{\hat{s}}_{n+1}\;\mathrm{or}\;{\hat{s}}_{n+1}=\frac{{\hat{s}}^{e}}{1+\frac{3\;G}{\sigma_{e}{|}_{n+1}}\left(\Delta \bar{\varepsilon }{}^{p}+\frac{A_{n+1}}{\Delta_{v}}\Delta \varepsilon_{v}^{p}\right)}.$$

The definition of the “direction tensor” $\hat{N}$ and Equation (A25b) show that ${\hat{N}}_{n+1}$ can be determined from the elastic predictor:

(A26) $${\hat{N}}_{n+1}=\frac{3}{2\;\sigma_{e}{|}_{n+1}}\;{\hat{s}}_{n+1}=\frac{3}{2}\frac{1}{\sqrt{\frac{3}{2}\;{\hat{s}}_{n+1}:{\hat{s}}_{n+1}}}\;{\hat{s}}_{n+1}=\frac{3}{2}\frac{1}{\sqrt{\frac{3}{2}\;{\hat{s}}^{e}:{\hat{s}}^{e}}}\;{\hat{s}}^{e}\equiv {\hat{N}}^{e}=\mathrm{know}.$$

Projection of (A25a) on ${\hat{N}}_{n+1}$ and the relationship $\hat{\sigma }:\hat{N}=\sigma_{e}$ lead to

$$\sigma_{e}{|}_{n+1}=\sigma_{e}^{e}-3\;G\left(\Delta \bar{\varepsilon }{}^{p}+\frac{A_{n+1}}{\Delta_{v}}\Delta \varepsilon_{v}^{p}\right),$$

where $\sigma_{e}^{e}={\hat{s}}^{e}:{\hat{N}}_{n+1}=\mathrm{know}$. The expression $A_{n+1}=A_{0}+\left(A_{1}/s_{a}^{*}\right)\sigma_{e}{|}_{n+1}$ is substituted in the last equation, which is then solved for $\sigma_{e}{|}_{n+1}$ to yield

(A27) $$\sigma_{e}{|}_{n+1}\left(\Delta \bar{\varepsilon }{}^{p},\Delta \varepsilon_{v}^{p}\right)=\frac{\sigma_{e}^{e}-3\;G\;\left(\Delta \bar{\varepsilon }{}^{p}+\frac{A_{0}}{\Delta_{v}}\;\Delta \varepsilon_{v}^{p}\right)}{1+\frac{3\;G}{s_{a}^{*}}\frac{A_{1}}{\Delta_{v}}\;\Delta \varepsilon_{v}^{p}}.$$

Substitution of Equation (A27) in the yield condition (A17) leads to

(A28) $$F\left(\Delta \bar{\varepsilon }{}^{p}\right)\equiv \frac{\sigma_{e}^{e}-3\;G\;\left(\Delta \bar{\varepsilon }{}^{p}+\frac{A_{0}}{\Delta_{v}}\;\Delta \varepsilon_{v}^{p}\right)}{1+\frac{3\;G}{s_{a}^{*}}\frac{A_{1}}{\Delta_{v}}\;\Delta \varepsilon_{v}^{p}}-{\tilde{\sigma }}_{0}\left(c_{n+1}^{\left(i\right)},{\bar{\varepsilon }}_{n+1}^{\left(i\right)}\right)=0,$$

where $\Delta \varepsilon_{v}^{p}$, $c_{n+1}^{\left(i\right)}$, and ${\bar{\varepsilon }}_{n+1}^{\left(i\right)}$ are all viewed as functions of $\Delta \bar{\varepsilon }{}^{p}$. $\Delta \bar{\varepsilon }{}^{p}$ is selected to be the primary unknown and Equation (A28) is the basic equation, in which $\left(\Delta \varepsilon_{v}^{p},{\bar{\varepsilon }}_{n+1}^{\left(i\right)},c_{n+1}^{\left(i\right)}\right)$ are defined in terms of the basic unknown $\Delta \bar{\varepsilon }{}^{p}$ by the equations:

(A29) $$\Delta \varepsilon_{v}^{p}\left(\Delta \bar{\varepsilon }{}^{p}\right)=\Delta_{v}\;c_{n}^{\left(a\right)}\;A_{f}{|}_{n}\;\alpha_{n}^{\left(a\right)}\;\Delta \bar{\varepsilon }{}^{p},$$

(A30) $${\bar{\varepsilon }}_{n+1}^{\left(i\right)}\left(\Delta \bar{\varepsilon }{}^{p}\right)={\bar{\varepsilon }}_{n}^{\left(i\right)}+\alpha_{n}^{\left(i\right)}\;\Delta \bar{\varepsilon }{}^{p},$$

(A31) $$c_{n+1}^{\left(1\right)}\left(\Delta \varepsilon_{v}^{p}\right)=c_{n}^{\left(1\right)}\exp\left(-\Delta \varepsilon_{v}^{p}\right),$$

(A32) $$c_{n+1}^{\left(2\right)}\left(\Delta \varepsilon_{v}^{p}\right)=c_{n}^{\left(2\right)}\exp\left(-\Delta \varepsilon_{v}^{p}\right),$$

(A33) $$c_{n+1}^{\left(3\right)}\left(\Delta \varepsilon_{v}^{p}\right)=f_{n+1}\left(\Delta \varepsilon_{v}^{p}\right)=f_{n}+\frac{\Delta \varepsilon_{v}^{p}}{\Delta_{v}},$$

(A34) $$c_{n+1}^{\left(4\right)}\left(\Delta \varepsilon_{v}^{p}\right)=c_{n+1}^{\left(a\right)}\left(\Delta \varepsilon_{v}^{p}\right)=1-\left(f_{n+1}+c_{n+1}^{\left(1\right)}+c_{n+1}^{\left(2\right)}\right).$$

The non-linear Equation (A28) for $\Delta \bar{\varepsilon }{}^{p}$ is solved using Newton’s method. After $\Delta \bar{\varepsilon }{}^{p}$ was found, $\left(\Delta \varepsilon_{v}^{p},{\bar{\varepsilon }}_{n+1}^{\left(i\right)},c_{n+1}^{\left(i\right)}\right)$ are calculated by using (A29)–(A34), $\sigma_{e}{|}_{n+1}$ is found from (A27), $A_{n+1}=A_{0}+\left(A_{1}/s_{a}^{*}\right)\;\sigma_{e}{|}_{n+1}$ is calculated, ${\hat{\sigma }}_{n+1}$ is determined from (A24), and the integration is completed with the calculation of

$$\sigma_{n+1}=R_{n+1}\;\cdot \;{\hat{\sigma }}_{n+1}\;\cdot \;R_{n+1}^{T}.$$

## Appendix B. Plane Stress Algorithm

In this appendix, a plane stress algorithm for the numerical integration of the constitutive equations is developed. In plane stress problems, the out-of-plane component of the deformation gradient is not defined kinematically and the algorithm described in Appendix A needs to be altered. The geometry analyzed is a thin plane disc of uniform thickness loaded in its plane. The mean plane of the disc coincides with the plane $X_{3}=0$, and the in-plane displacements are of the form

(A35) $$u_{1}=u_{1}\left(X_{1},X_{2}\right),\;u_{2}=u_{2}\left(X_{1},X_{2}\right),\;u_{3}=u_{3}\left(X_{3}\right).$$

The deformation gradient and the stress tensor, in isotropic materials, take the following form

(A36) $$\left[F\right]=\left[\begin{array}{ccc} F_{11} & F_{12} & 0\\ F_{21} & F_{22} & 0\\ 0 & 0 & F_{33} \end{array}\right]\;\mathrm{and}\;\left[\sigma \right]=\left[\begin{array}{ccc} \sigma_{11} & \sigma_{12} & 0\\ \sigma_{21} & \sigma_{22} & 0\\ 0 & 0 & 0 \end{array}\right],$$

or in compact form

(A37) $$F=F_{\alpha \beta }\;e_{\alpha }\;e_{\beta }+F_{33}\;e_{3}\;e_{3}\;\mathrm{and}\;\sigma =\sigma_{\alpha \beta }\;e_{\alpha }\;e_{\beta },$$

where $\left(e_{1},e_{2},e_{3}\right)$ are unit vectors along the coordinate axes and the summation convention on repeated Greek indices (α,β) is used. (α,β) take values in the range (1,2).

In finite strain problems, when the in-plane displacement field is inhomogeneous, the out-of-plane displacement and the corresponding thickness variation are functions of $\left(X_{1},X_{2}\right)$. It is assumed that, during material deformation, the resulting thickness variation is negligible, therefore the plane stress assumption is reasonable and (A36) are valid to a good approximation.

In plane stress problems, the method described in Appendix A needs to be modified since the out-of-plane component of the deformation gradient $F_{33}$ is not defined kinematically. The deformation gradient $\Delta F_{n+1}=R_{n+1}\;\cdot \;U_{n+1}$ associated with the current increment is written in the form

(A38) $$\left[\Delta F_{n+1}\right]=\left[\begin{array}{ccc} \Delta {\bar{F}}_{11} & \Delta {\bar{F}}_{12} & 0\\ \Delta {\bar{F}}_{21} & \Delta {\bar{F}}_{22} & 0\\ 0 & 0 & \Delta F_{33} \end{array}\right],$$

with $\Delta {\bar{F}}_{\alpha \beta }$(α,β=1,2) being the known in-plane components, and $\Delta F_{33}$ the unknown out-of-plane component. Likewise, the associated right stretch tensor $U_{n+1}$ and the orthogonal tensor $R_{n+1}$ from the polar decomposition of $\Delta F_{n+1}$ are expressed as

(A39) $$\left[U_{n+1}\right]=\left[\begin{array}{ccc} {\bar{U}}_{11} & {\bar{U}}_{12} & 0\\ {\bar{U}}_{21} & {\bar{U}}_{22} & 0\\ 0 & 0 & U_{33} \end{array}\right]\;\mathrm{and}\;\left[R_{n+1}\right]=\left[\begin{array}{ccc} \cos\bar{\theta } & -\sin\bar{\theta } & 0\\ \sin\bar{\theta } & \cos\bar{\theta } & 0\\ 0 & 0 & 1 \end{array}\right],$$

and the logarithmic strain tensor $E_{n+1}=\ln U_{n+1}$ associated with the increment is expressed as

(A40) $$\left[E_{n+1}\right]=\left[\begin{array}{ccc} \Delta {\bar{E}}_{11} & \Delta {\bar{E}}_{12} & 0\\ \Delta {\bar{E}}_{21} & \Delta {\bar{E}}_{22} & 0\\ 0 & 0 & \Delta E_{3} \end{array}\right],$$

where bared quantities $\Delta {\bar{E}}_{\alpha \beta }$ are known, and $\Delta E_{3}$ is the unknown out-of-plane component of $E_{n+1}$. It is worthy of note that the only difference with the method described in Appendix A is that the values of $\Delta F_{33}$ and $\Delta E_{3}$ are unknown when the process of the numerical integration starts. The plane stress condition is used to calculate the value of $\Delta E_{3}$:

(A41) $$\sigma_{33}{{|}_{n+1}={\hat{\sigma }}_{33}|}_{n+1}=e_{3}\;\cdot \;{\hat{\sigma }}_{n+1}\;\cdot \;e_{3}=0.$$

The logarithmic strain tensor associated with the increment is expressed as

(A42) $$E=\Delta \bar{E}+\Delta E_{3}\;a,\;\mathrm{with}\;a=e_{3}\;e_{3}=a^{\prime }+\frac{1}{3}\;\delta ,$$

where $\Delta \bar{E}=\Delta {\bar{E}}_{\alpha \beta }\;e_{a}\;e_{\beta }$ is the known in-plane part of E, and $a^{\prime }$ is the deviatoric part of a:

(A43) $$a^{\prime }=a-\frac{1}{3}\;a_{kk}\;\delta =-\frac{1}{3}\left(e_{1}\;e_{1}+e_{2}\;e_{2}-2\;e_{3}\;e_{3}\right).$$

Equations (A11)–(A20) are now expressed as follows:

(A44) $$\Delta E=\Delta E^{e}+\Delta E^{in}\;\Rightarrow \;\Delta E^{e}=\Delta E-\Delta E^{in},$$

(A45) $$\begin{array}{cc} {\hat{\sigma }}_{n+1} & =\sigma_{n}+L^{e}:\left(\Delta E-\Delta E^{in}\right)=\sigma_{n}+L^{e}:\left(\Delta \bar{E}+\Delta E_{3}\;a-\Delta E^{in}\right)=\\ & ={\bar{\sigma }}^{e}-L^{e}:\left(\Delta E^{in}-\Delta E_{3}\;a\right), \end{array}$$

(A46) $$c_{n+1}^{\left(1\right)}=c_{n}^{\left(1\right)}\exp\left(-\Delta \varepsilon_{v}^{p}\right),$$

(A47) $$c_{n+1}^{\left(2\right)}=c_{n}^{\left(2\right)}\exp\left(-\Delta \varepsilon_{v}^{p}\right),$$

(A48) $$f_{n+1}=f_{n}+\frac{\Delta \varepsilon_{v}}{\Delta_{v}},$$

(A49) $$c_{n+1}^{\left(a\right)}=1-\left(f_{n+1}+c_{n+1}^{\left(1\right)}+c_{n+1}^{\left(2\right)}\right),$$

(A50) $$\sigma_{e}{|}_{n+1}-{\tilde{\sigma }}_{0}\left(c_{n+1}^{\left(i\right)},{\bar{\varepsilon }}_{n+1}^{\left(i\right)}\right)=0,$$

(A51) $$\Delta E^{in}=\left(\Delta \bar{\varepsilon }{}^{p}+\frac{A_{n+1}}{\Delta_{v}}\Delta \varepsilon_{v}^{p}\right){\hat{N}}_{n+1}+\frac{1}{3}\;\Delta \varepsilon_{v}^{p}\;\delta ,\;A_{n+1}=A_{0}+\frac{A_{1}}{s_{a}^{*}}\sigma_{e}{|}_{n+1},$$

(A52) $$\Delta {\bar{\varepsilon }}^{\left(i\right)}=\alpha_{n}^{\left(i\right)}\;\Delta \bar{\varepsilon }{}^{p},$$

(A53) $$\Delta \varepsilon_{v}^{p}=\Delta_{v}\;c_{n}^{\left(a\right)}\;A_{f}{|}_{n}\;\alpha_{n}^{\left(a\right)}\;\Delta \bar{\varepsilon }{}^{p},$$

where bared quantities are known, and ${\bar{\sigma }}^{e}=\sigma_{n}+L^{e}:\Delta \bar{E}$ is the “elastic predictor” corresponding to the known part of ΔE.

First, the deviatoric and spherical parts of ${\hat{\sigma }}_{n+1}$ is calculated:

(A54) $${\hat{s}}_{n+1}={\bar{s}}^{e}-2\;G\left[{\left(\Delta E^{in}\right)}^{\prime }-\Delta E_{3}\;a^{\prime }\right],$$

(A55) $$\begin{array}{c} p_{n+1}=\frac{1}{3}\;{\hat{\sigma }}_{n+1}:\delta ={\bar{p}}^{e}-\kappa \left(\Delta E_{kk}^{in}-\Delta E_{3}\right), \end{array}$$

where ${\bar{p}}^{e}={\bar{\sigma }}_{kk}/3$, ${\bar{s}}^{e}={\bar{\sigma }}^{e}-{\bar{p}}^{e}\;\delta$, and ${\left(\Delta E^{in}\right)}^{\prime }$ is the deviatoric part of $\Delta E^{in}$. Combination of Equations (A51), (A54) and (A55) leads to

(A56) $${\hat{s}}_{n+1}={\bar{s}}^{e}-2\;G\left[\left(\Delta \bar{\varepsilon }{}^{p}+A_{n+1}\;\Delta f\right){\hat{N}}_{n+1}-\Delta E_{3}\;a^{\prime }\right],\;$$

(A57) $$p_{n+1}={\bar{p}}^{e}-\kappa \left(\Delta \varepsilon_{v}^{p}-\Delta E_{3}\right).$$

Use of the definition ${\hat{N}}_{n+1}=\frac{3}{2\;\sigma_{e}{|}_{n+1}}\;{\hat{s}}_{n+1}$ into (A56) leads to an expression for ${\hat{s}}_{n+1}$, which shows that ${\hat{s}}_{n+1}$ **and** ${\bar{s}}^{e}$ **are not proportional**:

(A58) $${\hat{s}}_{n+1}=\frac{{\bar{s}}^{e}+2\;G\;\Delta E_{3}\;a^{\prime }}{1+\frac{3\;G}{\sigma_{e}{|}_{n+1}}\left[\Delta \bar{\varepsilon }{}^{p}+\left(A_{0}+A_{1}\frac{\sigma_{e}{|}_{n+1}}{s_{a}^{*}}\right)\frac{\Delta \varepsilon_{v}^{p}}{\Delta_{v}}\right]}.$$

Last equation yields the following expression for $\sigma_{e}{|}_{n+1}$:

(A59) $${\left(\sigma_{e}{|}_{n+1}\right)}^{2}=\frac{3}{2}\;{\hat{s}}_{n+1}:{\hat{s}}_{n+1}=\frac{3}{2}\frac{\left({\bar{s}}^{e}+2\;G\;\Delta E_{3}\;a^{\prime }\right):\left({\bar{s}}^{e}+2\;G\;\Delta E_{3}\;a^{\prime }\right)}{{\left[1+{\frac{3\;G}{\sigma_{e}|}}_{n+1}\left(\Delta \bar{\varepsilon }{}^{p}+A_{n+1}\;\Delta f\right)\right]}^{2}}.$$

Since ${\bar{s}}^{e}:{\bar{s}}^{e}=\frac{2}{3}\;{\left({\bar{\sigma }}_{e}^{e}\right)}^{2}$, ${\bar{s}}^{e}:a^{\prime }={\bar{s}}^{e}:a={\bar{s}}_{33}^{e}$, and $a^{\prime }:a^{\prime }=\frac{2}{3}$, (A59) is expressed as

(A60) $${\left(\sigma_{e}{|}_{n+1}\right)}^{2}=\frac{{\left({\bar{\sigma }}_{e}^{e}\right)}^{2}+6\;G\;\Delta E_{3}\;{\bar{s}}_{33}^{e}+4\;G^{2}\;\Delta E_{3}^{2}}{{\left[1+\frac{3\;G}{\sigma_{e}{|}_{n+1}}\left(\Delta \bar{\varepsilon }{}^{p}+A_{n+1}\;\Delta f\right)\right]}^{2}}.$$

Next, the expression $A_{n+1}=A_{0}+\left(A_{1}/s_{a}^{*}\right)\sigma_{e}{|}_{n+1}$ is used and last equation is solved for $\sigma_{e}{|}_{n+1}$:

(A61) $$\sigma_{e}{|}_{n+1}=\frac{G}{1+\frac{3\;G}{s_{a}^{*}}\frac{A_{1}}{\Delta_{v}}\Delta \varepsilon_{v}^{p}}\left[F\left(\Delta E_{3}\right)-3\left(\Delta \bar{\varepsilon }{}^{p}+\frac{A_{0}}{\Delta_{v}}\Delta \varepsilon_{v}^{p}\right)\right],$$

where

$$F\left(\Delta E_{3}\right)=\sqrt{{\left(\frac{{\bar{\sigma }}_{e}^{e}}{G}\right)}^{2}+6\;\frac{{\bar{s}}_{33}^{e}}{G}\Delta E_{3}+4\;\Delta E_{3}^{2}}\;.$$

The quantities $\Delta \bar{\varepsilon }{}^{p}$ and $\Delta E_{3}$ are selected to be the basic unknowns of the problem. The primary equations for their determination are the yield condition and the plane stress condition ${\hat{\sigma }}_{33}{{|}_{n+1}={\hat{s}}_{33}|}_{n+1}+p_{n+1}=0$. Equations (A61) and (A57) for $\sigma_{e}{|}_{n+1}$ and $p_{n+1}$, are used together with (A58) to calculate ${\hat{s}}_{33}{|}_{n+1}$. Then, the basic equations of the problem take the form

(A62) $$\sigma_{e}{|}_{n+1}-{\tilde{\sigma }}_{0}\left(c_{n+1}^{\left(i\right)},{\bar{\varepsilon }}_{n+1}^{\left(i\right)}\right)=0,$$

(A63) $$\frac{{\bar{s}}_{33}^{e}+\frac{4}{3}G\;\Delta E_{3}}{1+3\;G\left[\frac{\Delta \bar{\varepsilon }{}^{p}}{\sigma_{e}{|}_{n+1}}+\left(\frac{A_{0}}{\sigma_{e}{|}_{n+1}}+\frac{A_{1}}{s_{a}^{*}}\right)\frac{\Delta \varepsilon_{v}^{p}}{\Delta_{v}}\right]}+{\bar{p}}^{e}-\kappa \left(\Delta \varepsilon_{v}^{p}-\Delta E_{3}\right)=0,$$

where $\left(\Delta \varepsilon_{v}^{p},c_{n+1}^{\left(i\right)},{\bar{\varepsilon }}_{n+1}^{\left(i\right)}\right)$ are all viewed as functions of $\Delta \bar{\varepsilon }{}^{p}$ via (A29)–(A34) and $\sigma_{e}{|}_{n+1}$ is defined in terms of $\Delta \bar{\varepsilon }{}^{p}$ and $\Delta E_{3}$ in (A61).

The system (A62)–(A63) for $\Delta \bar{\varepsilon }{}^{p}$ and $\Delta E_{3}$ is solved using Newton’s method. After $\Delta \bar{\varepsilon }{}^{p}$ and $\Delta E_{3}$ were found, $\left(\Delta \varepsilon_{v}^{p},{\bar{\varepsilon }}_{n+1}^{\left(i\right)},c_{n+1}^{\left(i\right)}\right)$ are calculated by using (A29)–(A34), $\sigma_{e}{|}_{n+1}$ is found from (A61), $A_{n+1}=A_{0}+\left(A_{1}/s_{a}^{*}\right)\;\sigma_{e}{|}_{n+1}$ is calculated, ${\hat{\sigma }}_{n+1}={\hat{s}}_{n+1}+p_{n+1}\;\delta$ is determined from (A58) and (A57), and the integration is completed with the calculation of

$$\sigma_{n+1}=R_{n+1}\;\cdot \;{\hat{\sigma }}_{n+1}\;\cdot \;R_{n+1}^{T}.$$
